# Biochip with multi-planar electrodes geometry for differentiation of non-spherical bioparticles in a microchannel

**DOI:** 10.1038/s41598-021-91109-2

**Published:** 2021-06-04

**Authors:** Amina Farooq, Nauman Z. Butt, Umer Hassan

**Affiliations:** 1 Department of Electrical and Computer Engineering, School of Engineering, Rutgers The State University of New Jersey, Piscataway, NJ USA; 2 Department of Electrical Engineering, Lahore University of Management Sciences, Lahore, Pakistan; 3 Global Health Institute, Rutgers The State University of New Jersey, New Brunswick, NJ USA

**Keywords:** Biotechnology, Bionanoelectronics, Microfluidics, Engineering, Electrical and electronic engineering

## Abstract

A biosensor capable of differentiating cells or other microparticles based on morphology finds significant biomedical applications. Examples may include morphological determination in the cellular division process, differentiation of bacterial cells, and cellular morphological variation in inflammation and cancer etc. Here, we present a novel integrated multi-planar microelectrodes geometry design that can distinguish a non-spherical individual particle flowing along a microchannel based on its electrical signature. We simulated multi-planar electrodes design in COMSOL Multiphysics and have shown that the changes in electrical field intensity corresponding to multiple particle morphologies can be distinguished. Our initial investigation has shown that top–bottom electrodes configuration produces significantly enhanced signal strength for a spherical particle as compared to co-planar configuration. Next, we integrated the co-planar and top–bottom configurations to develop a multi-planar microelectrode design capable of electrical impedance measurement at different spatial planes inside a microchannel by collecting multiple output signatures. We tested our integrated multi-planar electrode design with particles of different elliptical morphologies by gradually changing spherical particle dimensions to the non-spherical. The computed electrical signal ratio of non-spherical to spherical particle shows a very good correlation to predict the particle morphology. The biochip sensitivity is also found be independent of orientation of the particle flowing in the microchannel. Our integrated design will help develop the technology that will allow morphological analysis of various bioparticles in a microfluidic channel in the future.

## Introduction

Single-cell analysis is critically important in biological and clinical research for personalized disease diagnostics and therapeutics^1,2^. Characterization of individual cells offers detailed insights into their pathological condition and are therefore of huge interest to medical specialists^3^. Such data empowers a reliable diagnosis of diseases, infections, and biological threats^4^. Of different strategies proposed recently for single-cell analysis, cell impedance spectroscopy has pulled specific consideration because of the insights it provides into the mechanical, physical and biochemical properties of biological cells. Single cell impendence analysis helps medical experts with the methods not exclusively to identify the advancement of a disease during its very earliest phase, but also adequacy of treatment strategies^5,6^. Currently, there are several means of measurement that are typically used to detect cell by their size or volume. Importantly, the impedimetric cytometer based on coulter principle detects differential electrical signal of a biological cell under the influence of an externally applied electrical field^7–10^. This has many applications such as separating diseased cells from healthy cells, platelets from the whole blood, live from dead cells, budding yeast cells classification, and contaminants from the pure water, etc. Among various platforms, microfluidic impedimetric cytometry is established and frequently used in several medical applications due to low cost, label-free, high precision, and evident integration with on-chip electronic circuit for portable biosensors^11–13^.

The latest research highlights numerous numerical and exploratory examinations concerning different aspects of single-cell impedance spectroscopy such as cell morphology differentiation along with usual parameters such as size, volume, viability, and opacity^14–19^. Biological cells have variable shape and size like rod-shaped bacteria and biconcave shaped RBCs. Separation of non-spherical bioparticle is challenging because all existing single-cell methods rely upon the detection criteria based on the size and/or volume of a bioparticle i.e. diameter. Cell shape differentiation is a key physiological cellular condition that aids in medical analysis and microorganism growth parameter monitoring. For example, separation of live cells from dead cells through differences in electrical (i.e., conductivity and permittivity) and mechanical (i.e., size and shape) properties of cells^16^. Sickle cell anemia erythrocytes are rigid sickle-shaped RBCs due to abnormal hemoglobin polymerization which leads to cell rigidity and vasocculation^20^. Katsumoto et al. presented a sensor which can differentiate normal human RBCs from glutaraldehyde-treated (rigidified) as they were stretched by the high shear flow in a microchannel^2^. Asami designed spherical and elliptical shell models for investigating bacterial and yeast cell growth in cultures^1^. Kotb et al. successfully differentiated the normal human RBCs from the benzene exposed cells even at a very low level of exposure^21^. Malaria infection causes swelling of the erythrocyte with shape change from biconcave to spherical related to degradation of the cytoskeleton due to invasion of the parasite; studied in detail by Cowman and Crabb^22^. Ting Ye et al. developed two types of cell model for investigating the stretching deformation shape relaxation time for malaria infected RBCs^17^. The infected cells gradually lose their deformability and their ability to recover their original shape is greatly reduced with the maturation of the parasites. It is a complex dynamic process, accompanied by progressive changes in the shape, size, and mechanical properties of the cell. Furthermore, circulating tumor cells (CTCs) also exhibit highly variable shapes, including round, oval, elongated, and clusters. Non-round and multi-nucleate cells were sometimes observed^14,23^. Differentiation is based on large cancer cell size (e.g. Prostate cancer cell size ranged from 6.9—8.95 µm^14^ and lung tumor cells^15^). Similarly, CTCs derived from breast cancer have a median cell diameter of 13.1 µm^24^, and size-based method to isolate CTCs rely on their increased size^25^. Shashni et al. highlighted difference in surface deformity (roughness) between cancer cell lines (SP2/O cells and LLC cells) and normal cells (WBC and lymph node cell)^26^. Cell shape variation is predominant at some point in cell division or mitosis. Heterogeneous populations of budding yeasts have been analyzed and spherical, early-stage element and grown-up yeast were distinguished^27,28^. To characterize the physical morphology of single cells in parallel with the dielectric properties, Haandbæk et al. combines a microfluidic impedance cytometer with a high-speed camera^29^. The method used for classifying cells of the S. cerevisiae species as either single or budding cells based on the combined recording of optical images and impedance at a single-cell level. Apart from basic classification of single or budded cell, the device is unable to provide information about later stages of the yeast cell cycle based on impedance data. In addition, shape analysis of microorganism permits classification of microorganism in accordance with their type and might be used for microorganism anomalous growth diagnosis^4,30,31^. Moreover, Zeming and Ranjan separated the non-spherical bioparticle by using new array design in a microfluidic device^31^. In another paper, the authors developed a flow EIS device with constricted sensing area integrated with electrodes and used it to detect non-spherical particles because it provides higher signal-to-noise ratio^32^. Qiuet et.al., used the track etching method to create single pores in Polyethylene terephthalate (PET) and used the resistive-pulse technique as a sensitive method to distinguish between spherical and rod-shaped particles of various lengths^33^. Resistive pulse sensing (RPS) is based on the Coulter principle that have become increasingly popular over the last two decades while offering numerous biomedical applications encompassing environmental monitoring to diagnostics by enumerating metal ion and blood cells. RPS relies on a single particle passing through a pore and inducing a transient shift in device resistance, which is recorded as a resistive pulse, related to physical properties of object/particle. Maugi et al., designed a microfluidic sensor to detect particles below 5 μm while using polyurethane (PU) nanopores to identify the shape of individual nanoparticles in solution. However, nanorods with an aspect ratio less than 2 are indistinguishable from nanospheres in this setup^34,35^. Pollard et al. fabricated an RPS chip using additive manufacturing (AM) method with pores of 40 µm in diameter. The pulse shape shown was the characteristic of the channel dimensions^35^. Although, these findings are encouraging, the requirement that pore sizes must be close to the particles’ size being studied has disadvantages. First, requiring knowledge of predicted particle sizes restricts the technique's application to the analysis of uncertain or complex particle mixtures. Second, producing the smaller features involves specialized manufacturing techniques, and it's difficult to avoid being obstructed by larger analytes or particle aggregates.

The COMSOL Multiphysics has been used in the past for many biosensor projects. For instance, a COMSOL simulation on a single HeLa (human cervical epithelioid carcinoma) cell was developed to determine electrical properties (impedance, conductivity, and permittivity) to analyze the different cellular processes using a broad selection of voltage and frequency preconditions^3^. Sheng Hu et al. discussed in detail single cell dielectrophoretic (DEP) behavior in optoelectronic tweezers based on COMSOL ALE simulations^36^. Brazey et al. presented a comparison of two electrode geometries, square and star shape, via FEM simulation in COMSOL and then develop a microfluidic device for experimental validation^37^. The neural network approach proposed by Honrado et al. is based on 4-pair of electrodes with a trained supervised machine learning algorithm. Although, the study enabled the differentiation of particles size, speed, and cross-sectional position individually, it is unclear if the sensor would allow the differentiation of particles based on shape in reagent mixtures. However, in our proposed integrated design only two pairs of facing electrode with one additional central electrode provides both vertical and horizontal measurements allowing the particle’s shape differentiation^38^.

In this paper, we designed a novel multi-planar microelectrodes system for electrical differentiation of non-spherical bioparticles as they pass through a microchannel. Multi-planar microsensor is equipped with hybrid design of co-planar and top–bottom electrodes configurations. The passage of single particle through the channel result in producing electrical pulses with distinct features associated with different morphologies. Particle morphology is varied by gradually changing the spherical axis quotient from low to high value i.e. spherical to non-spherical (ellipsoidal) transformation. We demonstrated the efficiency of the morphology detection by flowing these particles through the channel and obtaining corresponding electrical signatures. The novelty of our device includes demonstration of efficient differentiation of particles with different morphology producing unique electrical characteristics in a novel multi-planar electrode configuration. Our presented configuration can potentially be used to explore several particles differentiations such as pathogens, RBCs, budding yeasts with enhanced sensitivity and efficiency, evading the constraint of spherical dimension from conventional methods.

## Methods

### Microfluidic channel with microelectrodes design

**Figure 1:**
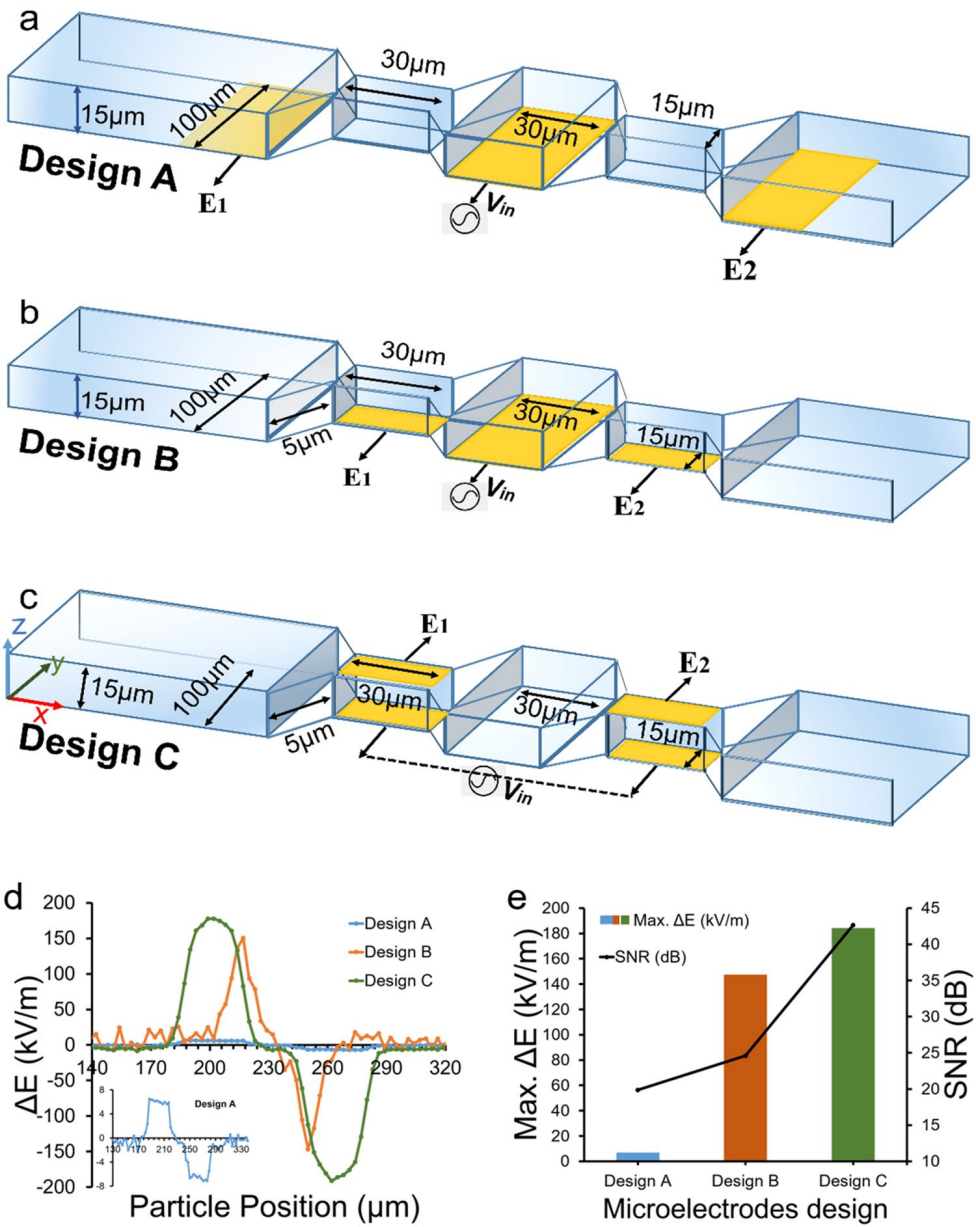
3-D view of all microfluidic channels with microelectrodes designs with geometrical dimensions, (**a**) Coplanar microelectrodes layout (Design A), (**b**) Modified coplanar microelectrode layout (Design B) with electrodes in narrow aperture, (**c**) Top bottom microelectrodes layout (Design C), (**d**,**e**) Electrical signal comparison in three microelectrode layouts. Design B, C outperformed Design A in signal strength. However, Design C produces highest signal strength and lowest noise. Figures (**a**–**c**) are drawn in Microsoft PowerPoint 365.

The single-cell analysis method is modeled utilizing the COMSOL Multiphysics simulation software. To create models for single cell microfluidic device, the AC/DC module of the COMSOL suite is used. Figure 1 shows a schematic 3-D diagram of the microfluidic channel with different configurations of microelectrodes employed in this study. The channel width, W is 100 µm and the channel height, H is 15 µm. Two sensing zone areas with dimensions of length × width × height as 30 µm × 15 µm × 15 µm are modelled within the microchannel. The focused region allows the electric field lines to be more concentrations in the sensing zone, thereby producing higher signal-to-noise ratio signals^23^. Microelectrode material is selected as Platinum with a thickness of 100 nm. The changes in the electric field intensity with the passage of particle will be measured at respective electrodes surface.

A conventional coplanar microelectrode design (Design A) is comprised of three co-planar electrodes of 100 µm width are separated by a gap of 35 µm and positioned at the bottom of microchannel outside both sensing zones as illustrated in Fig. 1a. To demonstrate the efficacy of sensing in the focused zone, we shifted the periphery electrodes to the bottom of the zone as illustrated in Fig. 1b (Design B). The middle bottom platinum electrode is excited by an AC voltage of 10 V and the corresponding changes in the electric field intensity is recorded between the exterior pair of platinum electrodes (E_1_ and E_2_). As the modeled particle sweeps through the microchannel, a differential bipolar pulse is produced whose amplitude is proportional to the diameter of the particle.

Top–bottom electrodes configuration design (Design C) is shown in Fig. 1c, where microelectrodes are placed exactly at the top and bottom planes of the sensing zones. The electrical field inside the microchannel is governed by the Laplace equation,

1 $$\Delta V=0$$

with the insulating boundary conditions at the channel walls and reservoirs, and with specified electrical potentials at the electrodes. The voltage potential is 10 V, and the functional form of the transient electrical field is the sine wave in this study. Since the Reynolds number is low, inertia terms can be neglected and the flow field inside the microchannel can be governed by the Stokes equation as, with the no-slip boundary conditions at the channel walls and with specified pressure values at the reservoirs. The input voltage signal is applied to the bottom electrodes and a differential signal (electric field intensity) is obtained from the top electrodes.

### Electric field intensity measurements

The electrical impedance measurement of biological entity is often accompanied by considering overall size or a volume occupied by a particle. In general, a biomolecule consists of a cell membrane, cytoplasm, and a nucleus in the middle. Each content of cell responded in a specific frequency range in impedimetric spectroscopy^39–44^. The electric conductivity of a particle is generally a value small as compared to the medium in which particle is suspended e.g. PBS solution. The resulting resistance between the electrodes increase considerably once a particle passes between them in the microchannel flow because of their electrical properties. Similarly, electrical permittivity of the particle and medium is frequency dependent and selection of appropriate frequency to be applied to the electrodes in critically important. The detectable frequency range for volume based cell differentiation is between 100 kHz and 1 MHz. The cell membrane shows a reactance peak for frequencies between 2–5 MHz. Cell interior conductance plays an important role in higher frequency range. Here, we focus on particle size and its shape and thus operating frequency range is low ~ 300 kHz. However, multi-frequency measurements for current proposed design can be explored for the study of various cellular conditions for specific biomedical applications.

We used a frequency of 300 kHz to electrically categorize particles based on their size and shape depending on the degree of difference between the resistances of the fluid properties and the particle membrane electrical parameters (conductivity and relative permittivity). The changes in the electric field intensity are measured at two sets of electrodes and a differential signal is proposed to minimize the common mode gain (a common cause of noise in electrical measurements). We found that the profile of ΔE signal (differential bipolar pulse) e.g., maximum ΔE peak, is affected not only by the size of the particle but also by its shape, position and the physical properties of the particle and the fluid properties. In this study, the position of the particle is fixed at the center of the detection volume and we sweep the particle along x-axis through both sensing regions of the microfluidic channel for measurements. We evaluate the shape and size of the particles by analyzing the profile of the ΔE signal as the particle passes through the sensing regions.

### COMSOL material specifications

Following material properties were used in selection of microparticles, fluid in the microchannel and electrode material. Biological cell/Microparticle: Microparticle is modelled as a biological cell with material specifications of 50 relative permittivity (ε_r_), 0.67 S/m electrical conductivity, 9 × 10^–4^ K^−1^ coefficient for thermal expansion (α), 1460 J/(kg-K) heat capacity at constant pressure (Cp), 970 kg/m^3^ density (ρ), 0.16 W/ (m*K) thermal conductivity at 25 °C (k), 7.5 × 10^8^ Pa elastic modulus (E), and 0.5 Poisson’s ratio (ν).

#### Phosphate Buffer Saline (PBS)

Microchannel geometry is filled with 1 × PBS solution at pH 7.4, which has specific material properties of 80 relative permittivity (ε_r_), 0.88 cP dynamic viscosity (µ) at 25 °C, 1.6 S/m electrical conductivity (σ), 4.186 × 10^3^ J/ (kg-K) heat capacity at constant pressure (Cp), 997 kg/m^3^ density (ρ), and 0.598 W/(m–K) thermal conductivity at 25 °C (k). The material properties used for the microfluidic design modelled in COMSOL Multiphysics 5.3a (conductivity and relative permittivity) employed in all simulations within this article have been summarized in Table S1.

#### Microelectrodes

We choose platinum material for the electrodes. Electrode material properties are selected as, 7 relative permittivity (ε_r_), 8.9 × 10^6^ S/m electrical conductivity, 8.8 × 10^–6^ K^−1^ coefficient for thermal expansion (α), 133 J/ (kg-K) heat capacity at constant pressure (Cp), 2.15 × 10^4^ kg/m^3^ density (ρ), 71.6 W/(m–K) thermal conductivity at 25 °C (k), 1.68 × 10^2^ MPa elastic modulus (E), and 0.38 Poisson’s ratio (ν). In COMSOL electrostatics module, PBS filled microchannel geometry was designated with zero charge and assigned with an initial value of 0 V. Charge was conserved through the constitutive relationship between electric field strength and relative permittivity:

$${\text{E}} = {\text{D}}/\varepsilon_{0} \varepsilon_{{\text{r}}}$$

where E is the electric field, D is dielectric constant, ε_r_ is relative permittivity, and ε_0_ is the relative permittivity of free space.

#### Mesh size

In our 3-D simulations, free tetrahedral meshing was used with ‘finer’ settings in COMSOL. Maximum element size for this setting is 2.86 µm, minimum element size of 0.25 µm and element growth rate of 1.45.

## Results and discussion

### Multiplex configurations of microelectrodes

Two most employed microelectrode configurations in the impedimetric microfluidic cytometer are coplanar and top–bottom electrode geometries^42,43,45–53^ In the conventional coplanar electrode design pair of electrodes have been placed at the bottom of microfluidic channel besides both sensing regions defined in microchannel. The electric potential has been applied to the middle bottom electrode at the center. When a particle passes between the set of microelectrodes through the sensing region, the change in differential electrical field distribution (E_1_–E_2_) has been recorded from the exterior electrodes as a bipolar pulse i.e. Design A (Fig. 1a). We modified this coplanar microelectrode configuration by shifting the exterior microelectrodes to the bottom of both focused sensing regions while keeping the middle electrode at the same position i.e. Design B (Fig. 1b). For top–bottom electrode configuration the electrodes are aligned at top and bottom parts of the microchannel at the focused regions i.e. Design C. The input signal is applied to pair of bottom electrodes and the differential electrical field data is obtained from top electrodes pair (Fig. 1c). Coplanar electrode configuration is prevalent due to its ease of fabrication; however, top–bottom electrode layout provides higher sensitivity due to uniform electric field distribution between electrodes across vertical axis of microfluidic channel.

We compared the performance of these electrode configurations by flowing a spherical particle with a diameter of 10 µm with electrical conductivity, σ = 0.67S/m, and relative permittivity, ε = 50. The bioparticle is swept through all three design layouts (Design A, B, & C) in COMSOL Multiphysics to yield the electrical signature for the spherical bioparticle. The resulting differential electrical field signals (E_1_–E_2_) for all design layouts are shown in Fig. 1d. A single bioparticle passing through the Design A layout can be seen in the inset of Fig. 1d. The signal strengths for the Design A, B, C configurations are 6.94 kV/m, 147.5 kV/m and 184.2 kV/m respectively. The standard deviation of the noise (σ) for the Design A, B, C configurations are 0.7 kV/m, 8.7 kV/m and 1.3 kV/m respectively. Design A produced much smaller signal strength as compared to Designs B and C, which is also evident by signal-to-noise (SNR) values shown in Supplementary Table 2. Design C performed the best with highest signal strength and lowest noise. The modification in existing coplanar electrode layout has proved to be substantially sensitive with enough SNR enabling better detection of 10 µm diameter particles. The peak signal strength values and the corresponding SNR values for all three design configurations are shown in Fig. 1d. In addition, we also straighten the center space in Design C as shown in Supplementary Figure S1. We performed the comparative analysis and flowed a 10 µm biological particle (σ = 0.67 S/m, ε = 50) across the microchannel between the electrodes. The results are show in Figure S2 and Table S3. Design C with broad center space resulted in higher signal strength, lower noise and higher SNR.

### Integrated multi-planar configuration of microelectrodes (IE)

**Figure 2:**
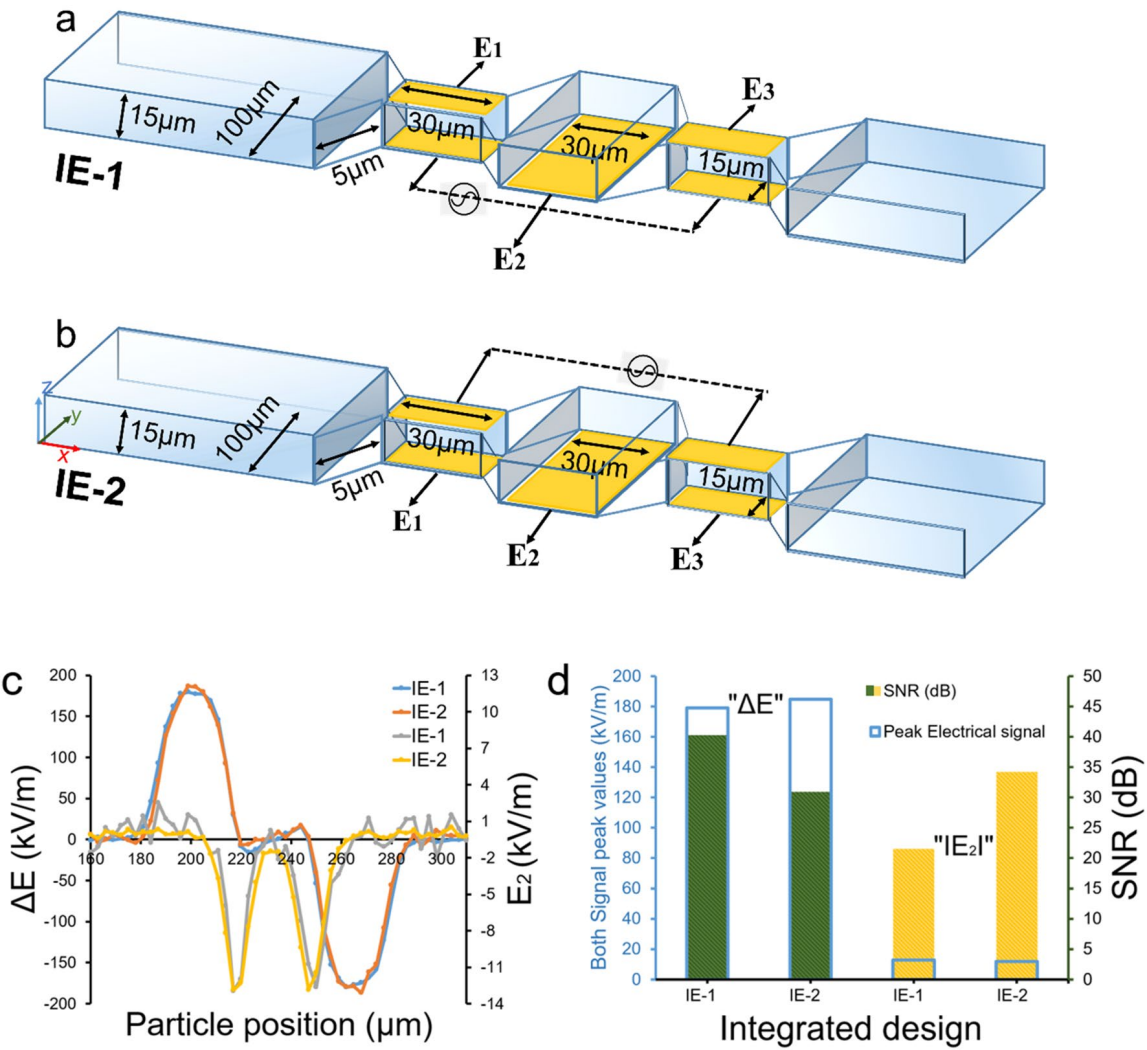
Detailed geometrical dimensions for integrated multi-planar designs of microelectrodes (IE-1 and IE-2) with microfluidic channel in 3-D view, (**a**,**b**) Integrated electrode designs with input signal and output signals; IE-1 (**a**) and IE-2 (**b**), (**c**) Output signals ΔE = E_1_–E_3_ (bipolar pulse) and E_2_ (unipolar pulse) from IE-1 and IE-2 relative to the particle position in the channel, (**d**) Comparison between the ΔE = E_1_–E_3_ and E_2_ signal peak values with respect to SNR in dB for IE-1 and IE-2. Figures (**a**,**b**) are drawn in Microsoft PowerPoint 365.

The profiles of electric field lines for co-planar and top–bottom design configurations are shown in Supplementary Fig. 3. Design C shows the uniform electric field lines across the vertical height. However, for Design B, the electric field lines are originating from middle to side electrodes in horizontal direction. We propose to combine the Design B and C configurations to develop a novel integrated multi-planar microelectrode configuration (IE) capable of morphological single cell analysis. The IE configuration was designed by integrating top–bottom and coplanar electrode layouts, allowing to perform two-fold single bioparticle sensing. We implemented IE configuration with two possible signal excitation designs which are shown in Fig. 2. The effect of grounding and applied signal to the electrodes pair were reversed and analyzed as illustrated in 3-D view of both designs. In IE-1 design, excitation signal is provided to bottom electrodes and multiple output signals are collected as ΔE = E_1_–E_3_ and E_2_, where ΔE is a differential bipolar signal and E_2_ is a non-differential unipolar signal (Fig. 2a). In IE-2 design, excitation signal is provided to top electrodes and multiple output signals are measured as ΔE = E_1_–E_3_ and E_2_ (Fig. 2b). Note that these two designs will generate a different electric field profile with respect to the middle bottom electrode. The electric field profiles are shown in Supplementary Fig. 4. We performed a comparative analysis and flowed a 10 μm particle across the microchannel over the electrodes. The resulting ΔE = E_1_–E_3_ and E_2_ signatures for both IE-1 and IE-2 designs are shown in Fig. 2c. In Fig. 2c,d we can observe that ΔE signal peak values for both IE-1 and IE-2 are approximately identical, however, the noise level of ΔE signal for IE-2 is much larger compared to IE-1. This results in much larger SNR for IE-1 (40.29 dB) compared to IE-2 (30.96 dB). The relevant values for noise, signal peak and SNR for ΔE signals can be found in Supplementary Table S4. Similarly, E_2_ signal peak values for both IE-1 and IE-2 are approximately identical, however, the noise level of E_2_ signal for IE-1 is much larger compared to IE-2. This results in much larger SNR for IE-2 (21.52 dB) compared to IE-1 (34.17 dB). The relevant values for noise, signal peak and SNR for E_2_ signals can be found in Supplementary Table S4. These unique differences of the noise between different designs can be explained by the electric field distribution profiles. For IE-1 design, there is a uniform electric field lines distribution from bottom electrode to top electrode, while there is non-uniform electric field distribution from bottom electrodes (where input signal is applied) to middle bottom electrode. Hence, we obtain ΔE with better SNR and E_2_ with less SNR. However, for IE-2 configuration, electric field vector starts from top electrodes and ends at all three bottom electrodes. This results into a more uniform electric field distribution throughout the sensing region. Therefore, it produces better SNR for both signals especially for E_2_. Further, note that the difference in both signals (ΔE and E_2_) SNR is much larger in IE-1 design as compared to IE-2.

### Sensitivity determination of the integrated design

**Figure 3:**
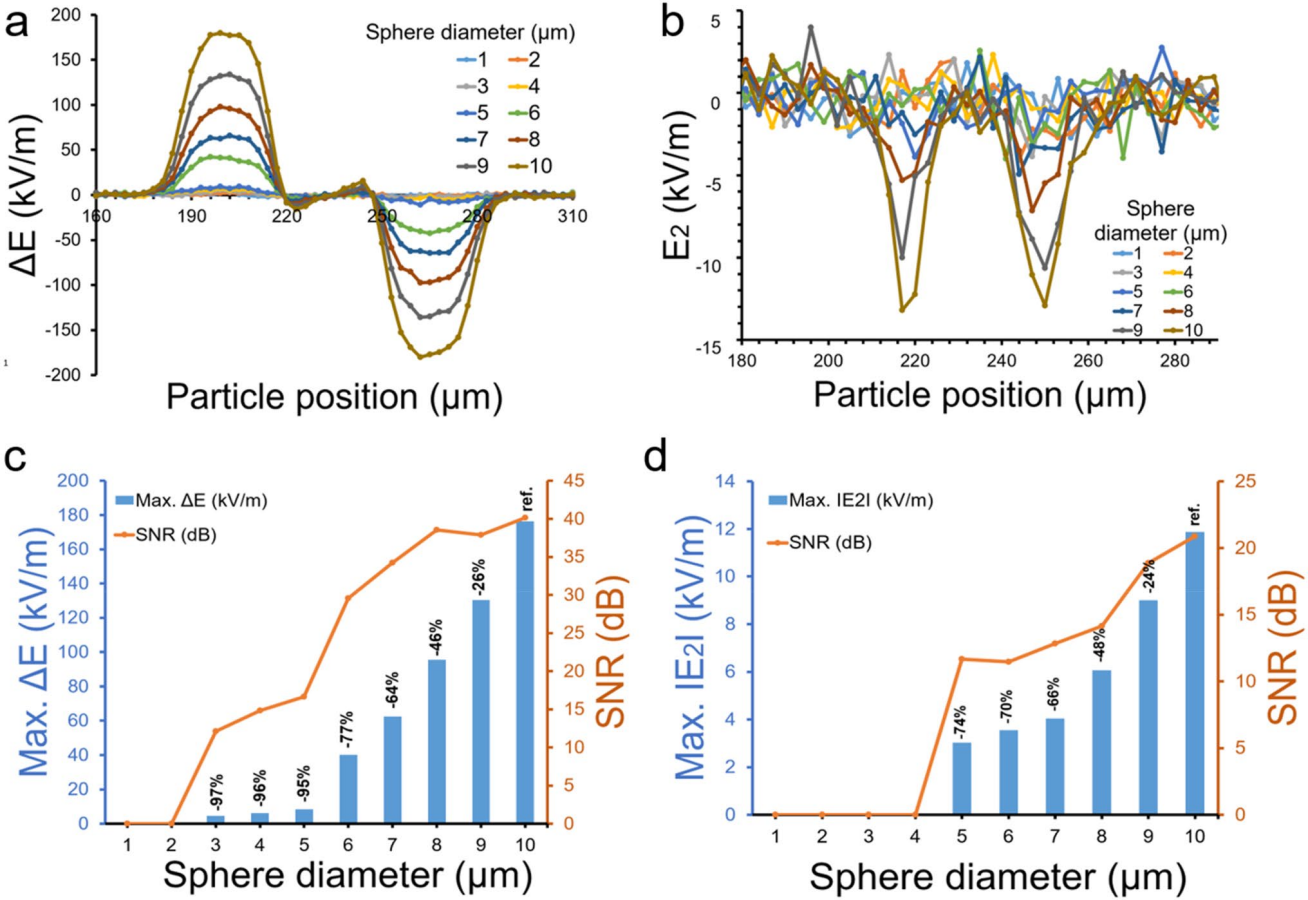
(**a**,**b**) Simulation of ΔE and E_2_ signals for spherical particle versus diameter increment from 1 to 10 in µm, (**c**,**d**) Comparison of maximum values of ΔE and │E_2_│and their resctive SNR in dB for spherical particle diameter ranges from 1 to 10 µm**.**

Next, we determined the sensitivity of device by simulating a spherical shape particle (modelled as a biological cell with electrical properties of ε = 50, σ = 0.67S/m). We analyzed the particle sizes (diameter) ranging from 1 to 10 μm. The simulation test determines the sensitivity and limit of detection (LOD) for the integrated designs when a single particle of varying size is traversed through the microchannel. Figure 3 illustrates particle of different diameters and their respective electrical signatures. Using IE-1 design configuration, ΔE and E_2_ signatures for 1–10 μm particles are shown in Fig. 3a,b respectively. Figure 3c shows exponential increase in the maximum ΔE values vs. particle diameter. For 10 μm particle SNR was found to be 40.2 dB. Reduction in the signals’ strength is 26% and 46% for a 9 μm and 8 μm respectively compared to a 10 μm particle. Similarly, a 7 μm, 6 μm and 5 μm particles’ resulting electrical signal percentage difference decreases up to 64, 77 and 95 percent respectively. The 5 μm and less diameter particles saw more than 96 percent decrease which is also visually evident from Fig. 3a. Similar analysis is also done for E_2_ signal and is shown in Fig. 3b,d. All the relevant values including ΔE, |E2|, noise and SNR are reported in Supplementary Table 5. The ΔE signal peak (Fig. S5a) for 1 μm and 2 μm particles and E_2_ signal negative peak (Fig. S5b) for 1-4 μm particles are not distinguishable from noise. Corresponding ΔE and E_2_ signals are shown in Supplementary Fig. 5. Limit of detection can be found by setting desired SNR value to be around 10 dB for the ∆E and E_2_ signature. For IE-1 design it comes to be 3 μm for ∆E signal and 5 μm for E_2_. Similarly, for IE-2 design configuration, ΔE and E_2_ signatures for 1–10 μm particles are shown in Supplementary Fig. S6a and Fig. S6b respectively. Fig. S6c shows a similar trend of exponential increase in the maximum ΔE values vs. particle diameter. For 10 μm particle SNR was found to be 31.1 dB and 34.8 dB for ΔE and |E_2_| signals respectively. Reduction in the signals’ compared to a 10 μm particle is shown in Fig. S4c and Fig. S6d for ΔE and |E_2_| signals respectively. The 4 μm and less diameter particles produces much smaller signal strengths and were indistinguishable from noise. The corresponding values including ΔE, |E_2_|, noise and SNR for IE-2 design are reported in Supplementary Table S6. The ΔE signal peak (Fig. S7a) and E_2_ signal negative (Fig. S7b) for 1 to 4 μm particles are not distinguishable from noise. Corresponding ΔE and E_2_ signals are shown in Supplementary Figure S7. Note that employing subsequent signal processing techniques (noise filters) can help improve the sensitivity and detection of these smaller particles in both IE-1 and IE-2 designs. Furthermore, SNR value, limit of detection and sensor sensitivity can be further improved by reducing the channel height, increasing signal strength, and with reduced electrodes spacing. If the microfluidic channel height is reduced to a level that is equivalent to the particle size under observation, the device's sensitivity will undoubtedly improve. However, fabrication of smaller dimensions on the other hand, may limit the robustness and ease of the fabrication processes. This current study aims to enhance the efficiency of existing unique microfluidic channel design that are simple and easy to build. Other research articles also studied the effect of electrode width, gap, and other geometrical parameters^23,51,54–56^. Wider electrodes allow for more current to pass through them, however, the fluidic aspect of width cannot be increased too much because it would cause vorticity in the dead volumes. The device with large electrode ~ 30–100 µm offers high sensitivity, repeatability and robustness as compared to smaller widths electrode design^23^. Further, detection of smaller size particles relies on multiple features including the geometry of the microfluidic sensing zone, electrodes dimensions and input voltage levels. The electrodes dimensions were chosen such that they are easy to fabricate and result in robust and repeatable sensors. Design A electrodes with similar dimensions and microfluidic geometry is demonstrated for biosensors in earlier studies^8–10^. We have extensively discussed the design strategies and methodologies in the Supplementary information. The benefits and drawbacks of different design parameters selection are mentioned the Supplementary Table S7.

### Spherical to non-spherical (or ellipsoidal) bioparticle discrimination

**Figure 4:**
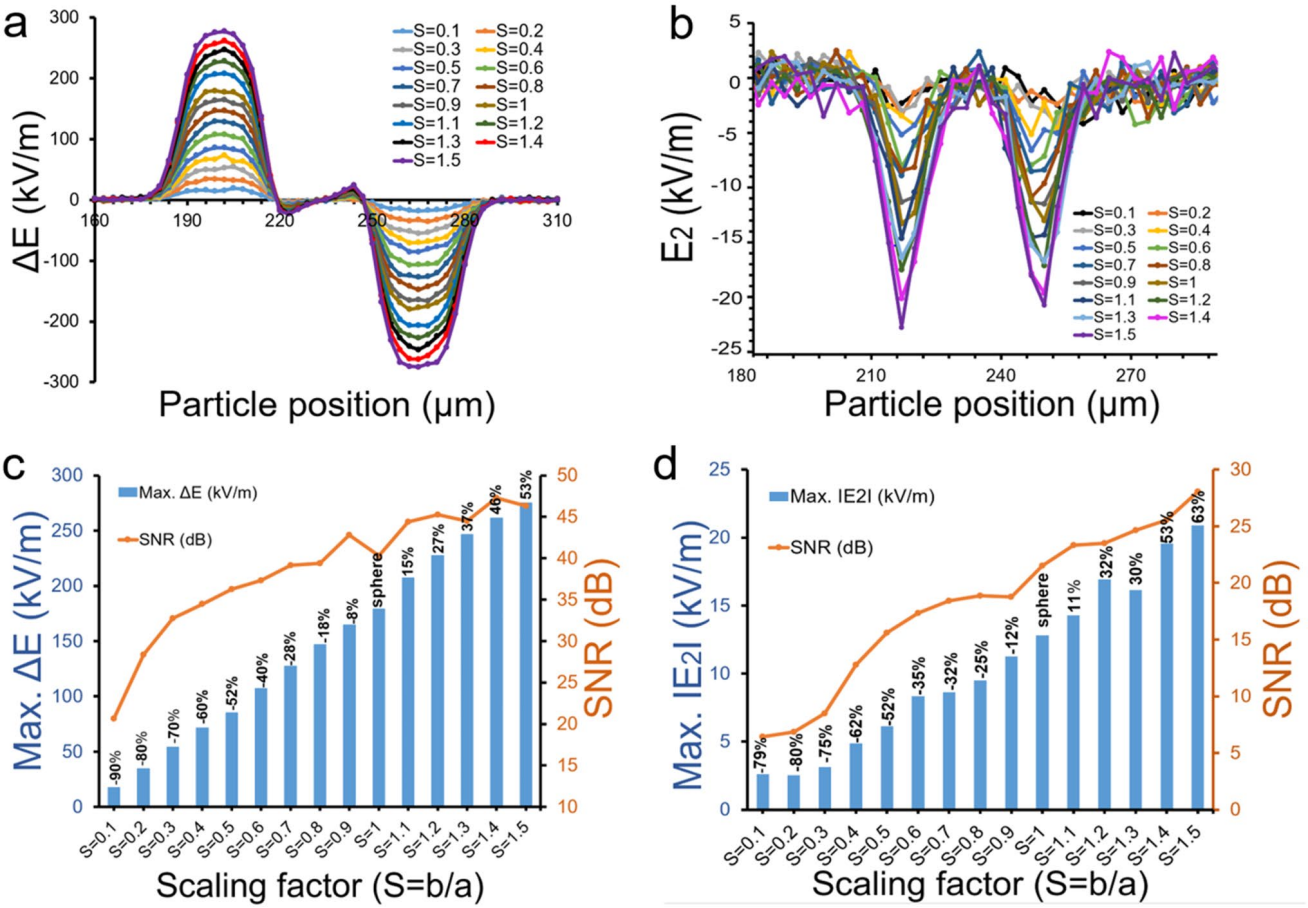
IE-1: Gradual change from spherical to non-spherical shape bioparticle i.e. S = 0.1–1.5 (with a = 5 µm remains fixed) for IE-1 design. (**a**,**b**) Simulation of ΔE and │E_2_│signals for particle axis ratio, S = b/a from 0.1 to 1.5. (**c**,**d**) Comparison of maximum values of ΔE and │E_2_│and their respective SNR in dB for particle shape ratio, S from 0.1 to 1.5. Plots (**a**–**d**) are drawn in Microsoft Excel 365.

Biological species come in many shapes and sizes, many are non-spherical in shape, e.g., disc-shaped red blood cells and rod-shaped bacteria. As a first step to distinguish the non-spherical particle we focused our effort on the ellipsoidal particles. We checked the sensitivity of our integrated multi-planar configurations (i.e. IE-1 and IE-2) for non-spherical particle detection. While doing so, we introduced a ratio that accounts for the non-spherical particle electrical signal maximum value with respect to the spherical shape particle electrical signature peak value due to the scaling factor. We define the scaling factor as a gradual change in spherical shape, mathematically;

2 $$S=b/a$$

where, a, and b are the axes lengths of a particle in x and y-axis directions. For a sphere a = b and S = 1. As one of these axes’ length changes, the spherical particle transforms into a non-spherical particle. This is illustrated in Supplementary Fig. S8. The scaling factor, S is set to 1 for an exact spherical shape (a = b) and subsequently varied between 0.1 to 1.5 for a given particle size with an increment of 0.1 as illustrated in a schematic diagram Fig. S8. In Eq. (2), S approaches to unity for a spherical shape. As S > 1, particle is enlarged in the vertical direction, however, for S < 1, particle shrinks in vertical axis and enlarge in horizontal axis. The scaling factor not only takes into consideration the perturbation of the electrical field between microelectrodes by the presence of the particle of various dimensions in the sensing region but also morphology changes in the particle shape. To test our system, we flowed particles (S = 0.1 to 1.5) through the microchannel in IE-1 and IE-2 designs. The computational results are given in Fig. 4a,b for both electrical signatures, ΔE and |E_2_| exhibiting a unique footprint for non-spherical shape particles in contrast to spherical particle (S = 1). There is a gradual signal variation manifested with the change in scaling factor, which corresponds to non-spherical shape detection. Notably there exists a symmetry between scaling factor and electrical signal peak values displayed in bar graphs in Fig. 4c,d, which also show the corresponding SNR values. A bar graph displayed both signal maximum value against particle shape ratio (i.e. scaling factor) varied from 0.1 to 1.5. A percentage difference is computed with respect to exact spherical shape (S = 1) to better account for signal sensitivity. As scaling factor increases or decreases the percentage of signal peak shifted accordingly, confirming sensitivity of electrical signature as shape changes slightly from spherical to non-spherical. Similarly, the computational results for IE-2 design are given in Supplementary Fig. S9 for both electrical signatures, ΔE and |E_2_|.

**Figure 5:**
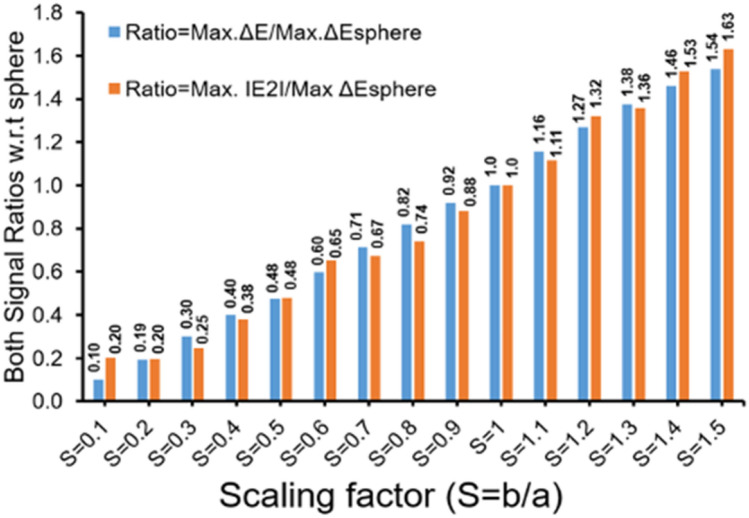
Maximum of signal ratios (ΔE and │E_2_│) with respect to sphere (S = b/a = 1) illustrates a gradual change from spherical to non-spherical shape particle. Scaling factor (S = b/a) increases from 0.1 to 1.5.

In further analysis, a ratio of ΔE signal peak values of non-spherical particle to spherical particle is computed for IE-1 design. Notably there is a linear correlation between scaling factor and signal ratio which us displayed in bar graphs in Fig. 5. A similar correlation can be observed for the ratio of |E_2_| signal peak values of spherical particle and non-spherical particle, represented by bar plots in Fig. 5. A similar analysis for IE-2 design configuration is shown in Supplementary Figure S10.

### Effect of particle orientation in the micro-channel

**Figure 6:**
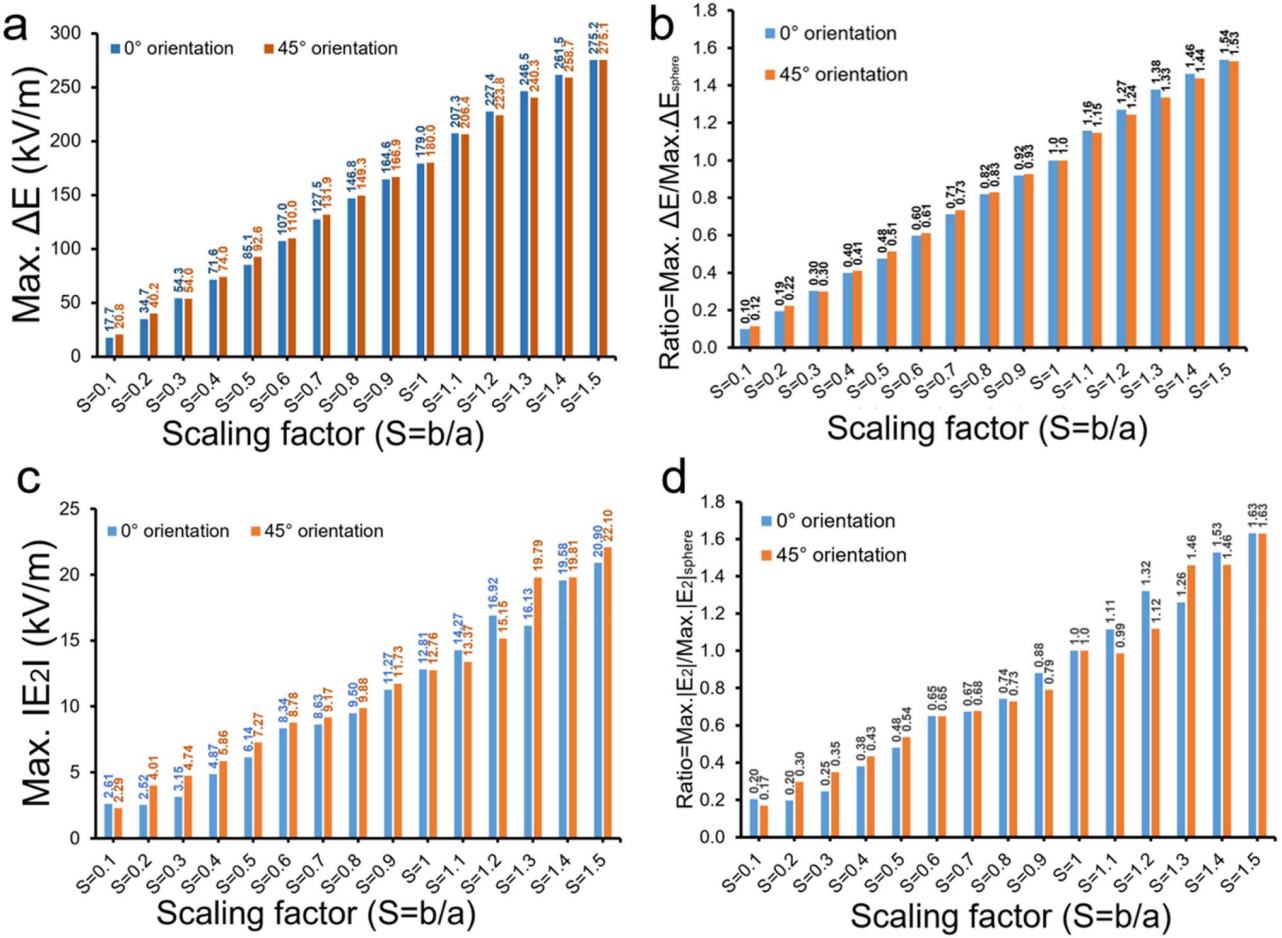
Comparison of spherical and non-spherical particle signal variation with respect to orientation (0-degree and 45-degree) within integrated design IE-1. (**a**) Maximum of ΔE with respect to scaling factor at two different orientations of particle position, (**b**) Comparison of ration of maximum value of ΔE of non-spherical particle (S = 0.1 to 1.5) to spherical particle for both orientations, (**c**,**d**) Comparsion of maximum |E_2_| signal and |E_2_| ratio at different sacling factors for both orientations.

Particle orientation in the microchannel is an important consideration since it will impact the particle position in the electric field and resulting electrical signatures. To test the dependence of our IE-1 design on the particle orientation, we compared the simulation results for the particles orientated at 45-degree angle with respect to x-axis (i.e. representing maximum effect of orientation). The particles were flowed through the microfluidic channel and ΔE and |E_2_| were found. Electrical signals were obatined for particle positioned at two different orientation (0° and 45°) within microchannel. ΔE and |E_2_| signatures for IE-1 configuration for both particle orientations are shown in Supplementary Figure S11. Comparison of both orientations for IE-1 configuration is given in Fig. 6. Maximum of ΔE with respect to scaling factor at both different orientations of particle position is shown in Fig. 6a, while Fig. 6b shows the computed ratios of ΔE signal peak values of non-spherical particle (S is varied from 0.1 to 1.5) to spherical particle at both orientations. Comparative analysis shows a small difference because of varied particle orientations in the microchannel. Similarly, maximum of |E_2_| signal with respect to scaling factor at both different orientations of particle position is shown in Fig. 6c, while Fig. 6d shows the computed ratios of |E_2_| signal peak values of non-spherical particle to spherical particle at both orientations. We also performed a similar orientation analysis for IE-2 design. ΔE and |E_2_| signatures for IE-2 configuration for both particle orientations are shown in Supplementary Fig. S12. Similarly, comparison of ΔE and |E_2_| with scaling factor for both orientations is shown in Supplementary Fig. S13a,c. However, comparison of ΔE and |E_2_| ratios of non-spherical to spherical particle for both orientations is shown in Supplementary Fig. S13b,d. Both IE-1 and IE-2 designs were found be independent of orientations of the particle flowing in the microchannel.

### Relationship of mesh size and noise in the simulations

Our COMSOL model can be described by partial differential equations (PDE) and the solution to the mathematical model is approximated through the finite element method (FEM) in COMSOL Multiphysics software. FEM is used to discretize the problem and divide the model geometry into tiny elements. This leads to different levels of noise and can be improved by mesh adaptation. By selecting high density mesh distribution, we can decrease errors in physical results and yield more accurate results. While building a mesh, several factors should be taken into consideration, which can be addressed in various features functionality available in the software. This include choosing a mesh sequence type either by an automated process or a customized meshing by manual settings. We have chosen an automated physics-controlled meshing sequence which offers different mesh settings with the options of ‘normal’, ‘fine’, ‘finer’, ‘extra fine’, and ‘extremely fine’. Other lower resolution mesh options are also included e.g., ‘coarse’, however, in microstructures, these settings give unsatisfactory results with much higher noise.

To investigate the relationship between noise and mesh size, we have simulated a 10 µm spherical particle using our integrated design (IE-1) with four mesh settings in order to highlight meshing effects on signal strength and signal to noise ratio (SNR). We have added the corresponding Supplementary Figure S14 and Table S8 and Table S9 with all meshing parameters listed. Although ‘extremely fine’ mesh setting provide higher element quality, however, time and space complexity becomes much higher for it to be used. Both ‘finer’ and ‘extra fine’ mesh settings give good electric signal strengths and signal to noise ratios as compared to ‘fine’ mesh setting. Supplementary Figure S14c, shows the peak-to-peak simulation noise at these three mesh settings and its effect on overall signal to noise ratio is shown in Supplementary Figure S14b. It should be noted that once a particular mesh size is selected, the noise of the electrical signals defined earlier depends on the electrode designs and configurations.

### Differentiation of particles with fixed volumes

In the next simulation study, we kept the volume of different particles’ approximately the same with each other, and adjusted their geometrical parameters. We simulated the spherical, and elliptical particle in horizontal and vertical orientations as shown in Supplementary Fig. 15. We flowed these particles through our counter and the results are tabulated in Supplementary Table S10. We observed that the particles with the same width (i.e., sphere and ellipse-x), the pulse width half maximum (FWHM) and pulse amplitude can be used to discriminate elliptical and spherical particle. Both signals ΔE & |E_2_| confirms the particle shape differentiation along with their size. ΔE can be used for differentaiting elliptical particle of same volume as spherical one has higher amplitude while smaller peak value for |E_2_| signal differentiates the elliptical shape. When the particles’ volume is same and shape is different, we propose an ‘R’ value defined as the ratio of FWHM and maximum value of ΔE obatined. Two different R values are calculated based on ΔE and |E_2_| signatures i.e., (i) R_A_ = FWHM (ΔE) / max (ΔE), and (ii) R_B_ = FWHM (E_2_) / max (|E_2_|). R_B_ is significantly different for elliptical particle as compared to spherical one. R value can be used as a discriminative parameter with pulse width or amplitude. Now, considering a case when the particles length were approximately the same (i.e., sphere and ellipse-y), the pulse amplitudes ΔE and R_A_ parameters can easily be used to discriminate the particle shape. In comparsion, we found that for fixed volume particle differentiation, |E_2_| signal is less discriminative compared to ΔE due to non-uniform electric field distribution in coplanar electrodes geometry. Hence an integrated design which provides both electric signals simultaneously helps in better shape differentiation.

In comparison, similar analysis and R value can also be used to differentiate particles if the particles’ volume is not kept same. To further investigate this, we kept length of the particles’ fixed i.e., 10 µm and changes the other parameter to vary the volume and shape. The width to length ratio is varied from a lower value to a maximum one, S = 0.1 to 1.5, which changes the volume of the particle along with particles shape, which gradually changes from elliptical to spherical. We flowed these particles through our counter and the results are tabulated in Supplementary Table 11. We observed that the particles with the same length have almost same pulse width half maximum (FWHM) value, however, the pulse amplitude or the R value can be used to discriminate particle shape (ellipse or sphere). Both signals ΔE and |E_2_| confirms the particle shape differentiation as the width of the particle changes. ΔE and |E_2_| for elliptical particles of same length but smaller widths compared to spherical, has smaller pulse amplitudes. Further, with different shape and volume particles, we calculated R value. We found that both R_A_ and R_B_ are significantly different when the particles volume and shape is different. Importantly, when the particles’ length are approximately the same the impedance pulse amplitude, ΔE, and R_A_ can be used to discriminate the particle shape. Our system’s ability to distinguish mixture of particles is discussed in Supplementary Table S12, a special case is considered with mixture of particles containing 2 and 4 µm spherical particles and 3 µm (length or width) elliptical particle using IE-1 design. We observed that for particles with different width, the pulse full width half maximum (FWHM) and pulse amplitude can be used to discriminate elliptical and spherical shapes. R_A_ value can also be used to differentiate particles if the particles’ volume is not kept same. It is defined as the ratio of FWHM and maximum value of ΔE obatined. We found that both R_A_ and ΔE are significantly different when the particles volume and shape is different. Importantly, when the particles’ length are approximately comparable, the impedance pulse amplitude, ΔE, and R_A_ can be used to discriminate the particle shape. In case of a complex mixture, e.g., when cell is dividing their total volume increases with enlargement in one direction^28,57^. So, we can consider the case of spherical cell comparison with ellipse-y that has S = 1.5 in supplementary Table S11. Similarly, in case if cells are dead then their overall volume decreases^58^ which can be considered as like the case of ellipse-x with S = 0.1 and 0.5. Therefore, an integrated design proposed in this study provides a good approximation for particle shape differentiation along with particle size.

## Discussion and conclusion

In a two-electrode system when a voltage is applied at one electrode surface and ground the opposite electrode, it generates the resulting electric field distribution between them. When a particle passes through the sensing zone, it disrupts the electric field distribution, which is recorded as change in electric field value and data is acquired from the electrode surface. Addition of a third middle electrode to the single pair is used for a differential sensing, which provides common mode error reductions to improve signal to noise ratio (SNR). Practically in real world situation this change in electric field will be reflected as the change in impedance which can easily be acquired via Wheatstone bridge i.e., by measuring the potential drop across a resistor, R as shown in Figure S16b. Figures S16 a & c further clarifies input and output obtained from electrodes’ surface in a 3-electrode layout and in our modified integrated multiplanar electrode configuration (IE-1). Furthermore, the bipolar pulse obtained from the differential signal ΔE or ΔV = V_1_-V_2_, represents footprints of a particle or a cell which can further be translated into current or amplified as required.

Our proposed sensor design presents a unique direction to differentiate the morphology of the particles flowing in the microchannels. This computational study has demonstrated that integrating microelectrodes in coplanar configuration with top–bottom electrode configuration will increase the sensor’s sensitivity and SNR. It results in the effective electrical differentiation of the particles’ size and morphology. We demonstrated non-spherical bioparticle discrimination by using our proposed integrated multi-planar configuration of microelectrodes owing to their unique changes in the electrical signature. The separation is accomplished using a uniform electric field which was generated by an integrated geometry of a microelectrodes. Moreover, sensor sensitivity is further investigated by simulating spherical and elliptical shaped particles. A microsensor consisting of a constricted microchannel and a modified microelectrode configuration is proposed and evaluated in order to measure the transformation of a single spherical shape bioparticle into elliptical by analyzing the changes in the electric field intensity. A strong correlation was observed between the peak amplitude of signals (ΔE and |E_2_|) of non-spherical (i.e., elliptical) particle axis ratio (i.e., scaling factor) to peak amplitude of signal for a spherical particle. Two different integrated designs i.e., IE-1 and IE-2 were proposed. Both designs provided good signal strength, SNR value and were able to differentiate spherical vs. non spherical particles. In comparison, IE-1 design produced better results because of lower limit of detection. However, it should be noted that the limit of detection and the sensor sensitivity can be further improved by employed post-processing data filtering techniques to remove noise. Furthermore, reducing the channel height or increasing the input electric field strength will further improve the accuracy in differentiation smaller particles.

Particle rotation while flowing in the microfluidic channels is an important consideration since it will impact the particle position in the electric field and resulting electrical signatures. In various practical experimental conditions e.g., microparticles of elliptical shape (such as E. coli) won’t always align themselves parallel to the sensing zone while flowing through the microchannel. Non-spherical microparticles may cross the sensing zone in different orientations e.g., vertical, or diagonal positions. Such examples have been reported earlier in many cases^44,59–64^. The variation in the orientation for particle flow depends on many factors including flow rate, pressure, biasing, dilution factor, hydrodynamic forces and other manual sample handling e.g., 3D asymmetrical inlet holes in microfluidic devices. Therefore we analyzed two most possible orientations i.e., 0° and 45° that particles can take while flowing through the channel as an in-depth computational study. We evaluated the dependence on the particle orientation in the microchannel at 0° and 45° positions along the horizontal axis. Both IE-1 and IE-2 designs showed small variations in the corresponding electrical signatures. In future, collection of multiple features of electrical signatures ΔE and |E_2_| can be used to develop multi-variate computational models based on machine learning techniques to improve the sensor accuracy^53^. Table S13 details a comparative analysis of our study with earlier reports on the similar topic. We compared the specific features including size, shape and cell classification in single cell cytometry in the Table S13 and list the limitations. Our integrated design has been proven to be more effective in the classification of non-spherical bioparticles compared to existing techniques by using the ellipsoidal shape as proof-of-concepts for the potential differentiation of the various non-spherical biological entities, for instance blood cells, bacteria, and others entities in future.

## Supplementary Information

Supplementary Information.
