# Supplementary material for: Biochip with multi-planar electrodes geometry for differentiation of non-spherical bioparticles in a microchannel
Source: Sci Rep. 2021 Jun 4;11:11880. doi: 10.1038/s41598-021-91109-2 (PMC8178319; doi:10.1038/s41598-021-91109-2)
Supplement: Supplementary file 1 — Supplementary Information. [file 41598_2021_91109_MOESM1_ESM.pdf]

SUPPLEMENTARY INFORMATION

Biochip with Multi-planar Electrodes Geometry for Differentiation of Non-Spherical Bioparticles in a Microchannel

Authors: Amina Farooq[1,2], Nauman Z. Butt[2], Umer Hassan*[1,3]

[1] Department of Electrical and Computer Engineering, School of Engineering, Rutgers The State University of New Jersey, Piscataway, NJ, USA

[2] Department of Electrical Engineering, Lahore University of Management Sciences, Lahore. Pakistan

[3] Global Health Institute, Rutgers The State University of New Jersey, New Brunswick, NJ, USA

*Corresponding Author: umer.hassan@rutgers.edu; Tel (848) 445-2164

Supplementary Text

Device design strategy & methodology: The device dimensions were selected to improve the chip's sensitivity in the sub micrometer range while also allowing it to detect larger particles (up to 10 µm). This sensing region (30 µm × 15 µm × 15µm) selection is large enough to facilitate particles up to 14 µm diameter without clogging, and compact enough to ensure that the cells passed through the electrodes in a single file with little difference in passage height, ensuring accurate cell enumeration. The effect of geometrical parameters on parallel and coplanar electrodes configuration is discussed in detail in numerous research articles [1-5]. For example, T. Sun et.al., analytically solved the electrical field distribution in two widely used electrodes set up for impedimetric sensing in microfluidics, coplanar and parallel configuration [3]. Influence of constricted channel geometrical parameters on device sensitivity is studied in detail by J. Cottet et.al., [2] and C.H. Clausen et.al., [1]. The electric field distribution in a parallel electrode configuration is determined by two geometrical parameters: electrode length and channel height. Increasing the electrode length extends the quasi-homogeneous electric field area between parallel configuration, and the magnitude of the electric field in the entire microchannel rises. And increasing the channel height decreases the magnitude of the field in the quasi-homogeneous region. However, the field magnitude outside the quasi-homogeneous region increases. The electric field distribution in a coplanar electrode configuration is determined by three geometrical parameters: electrode width, channel height, and electrode gap. The area of quasi-homogeneous electric field is unaffected by decreasing electrode length (in the center). Increasing the electrode difference results in a larger area of quasi-homogeneous field in the center (but at a lower magnitude) and a larger electric field outside the inter-electrode zone.

Elongation of the electrodes along the channel direction (~100 µm) results in a higher current between the electrodes (Supplementary Figure S1-2). This width of electrode was chosen as the maximum pulse amplitude detection, a compromise between the maximum sensitivity and fluidic constraints [2]. However, it is not beneficial to increase the length of the electrodes (~30 µm) significantly since this will result in inconveniently long particle transition times. The length of the electrodes was fixed at 30 µm for both the integrated optimized design, which was found to be a good compromise between signal strength and transition length. The distance between the coplanar electrodes was set to 5 µm in both layouts, offers large confinement of the electric field lines [2], which provides high sensitivity while keeping a detection volume large enough for bigger particles. The counting electrodes dimensions are chosen to ensure adequate resolution and to create a sensing region volume of ~7 pL (30 µm × 15 µm × 15µm), both to ensure coincident events would not occur the device is capable of single particle analysis up to a concentration of approximately 4-11 × 10^3 cells per µL biological cells, a physiological range found in humans [1-5].

Supplementary Tables

Table S1: Microfluidic channel specifications used in COMSOL Multiphysics 5.3a with input frequency 300 kHz and input voltage is set to 10 V

Geometrical material	Material Property	Relative Permittivity	Electrical Conductivity (S/m)
**Channel**	1x PBS	80	1.6
**Electrodes**	Platinum	7	8.9×10^6
**Particle**	Biological cell	50	0.67

Table S2: Significant Increase in signal amplitude for 10μm diameter spherical bioparticle in Design B as compared to Design A and stand very close to Design C values. As expected, the peak differential signal strength induced by an operational voltage of 10 V within sensing region increases in modified design as compared to conventional coplanar configuration as good as top-bottom layout.

Electrode Design	σ_{noise} (kV/m)	Max. ΔE (kV/m)	SNR (dB)	Percentage difference (%)
Design A	0.7	6.94	19.86	-----
Design B	8.7	147.5	24.59	2024
Design C	1.3	184.2	42.66	2551

Table S3: Simulations results for a comparative analysis for two different configurations of Design C with a straight and a broader center space.

Constricted channel Central Space	Avg. Max. ΔE (kV/m)	σ_{noise} (kV/m)	SNR (dB)
Broad	187.2	5.46	30.69
Narrow & Straight	120.1	7.94	23.59

Table S4. Signal values and their SNR in dB in two different integrated designs IE-1 and IE-2.

Design	Avg. Max. ΔE (kV/m)	σ_{noise} (kV/m)	SNR (dB)	Avg. Max. $\lvert E_2 \rvert$ (kV/m)	σ_{noise} (kV/m)	SNR (dB)
IE-1	179	1.73	40.29	12.8	1.07	21.52
IE-2	184	5.22	30.96	11.8	0.23	34.17

Table S5: Average values for both signal ΔE and $|E_2|$ in kV/m along with SNR in dB and σ_{noise} in kV/m for spherical shape particle versus diameter increment from 1 to 10 in µm for IE-1 design.

Sphere Diameter (µm)	Avg. Max. ΔE (kV/m)	σ_{noise} (kV/m)	SNR (dB)	Avg. Max. $|E_2|$ (kV/m)	σ_{noise} (kV/m)	SNR (dB)
1	In-distinguishable	0.89	----	In-distinguishable	1.30	----
2	In-distinguishable	1.02	----	In-distinguishable	1.05	----
3	4.61	1.14	12.1	In-distinguishable	0.79	----
4	6.07	1.10	14.8	In-distinguishable	1.07	----
5	8.31	1.22	16.6	3.02	0.79	11.6
6	40.03	1.33	29.6	3.56	0.95	11.4
7	62.42	1.21	34.2	4.04	0.92	12.8
8	95.44	1.12	38.5	6.07	1.19	14.1
9	130.38	1.66	37.9	8.99	1.02	18.8
10	176.27	1.73	40.2	11.87	1.07	20.8

Table S6: Average values for both signal ΔE and $|E_2|$ in kV/m along with SNR in dB and σ_{noise} in kV/m for spherical shape particle versus diameter increment from 1 to 10 in μm for IE-2 design.

Sphere Diameter (μm)	Avg. Max. ΔE (kV/m)	σ_{noise} (kV/m)	SNR (dB)	Avg. Max. $\|E_2\|$ (kV/m)	σ_{noise} (kV/m)	SNR (dB)
1	In-distinguishable	3.29	----	In-distinguishable	0.21	----
2	In-distinguishable	3.78	----	In-distinguishable	0.21	----
3	In-distinguishable	4.42	----	In-distinguishable	0.24	----
4	In-distinguishable	3.69	----	In-distinguishable	0.22	----
5	12.60	4.13	9.7	0.88	0.24	11.08
6	42.33	4.24	20.0	2.66	0.21	21.7
7	68.46	4.17	24.3	4.14	0.16	28.0
8	98.68	4.40	27.0	6.62	0.20	30.2
9	134.28	5.57	27.6	9.19	0.23	31.8
10	188.45	5.22	31.1	12.88	0.23	34.8

Supplementary Table S7: Microfluidic channel and microelectrodes dimensional parameters selection strategy.

Device Geometrical Parameters	Dimensions (µm)	Pros	Cons	ref
Parallel Electrode length = Sensing region length	30	The quasi-homogeneous electric field region becomes wider and the magnitude of the electric field in the entire sensing region increases.	Overall device size increases	[3]
Parallel electrode gap = Microchannel height	15	High magnitude of the electric field in the quasi-homogeneous region with small magnitude outside it.<br>Accommodates sensing of large particles, aggregates and minimize clogging issues.	Limited SNR for smaller particles.	[3]
Gap between parallel electrodes = Mid-region length	45	Maximum magnitude of change in impedance induced	long particle transition time	[2, 4]
Coplanar electrode length	30	More current lines added by long electrodes, and decreasing the length does not much influence the region of quasi-homogeneous electric field	long particle transition time for parallel set up	[2, 3]
Gap between coplanar electrodes	5	Leads to a larger magnitude of quasi-homogeneous field in the center. Smaller particle transition time.	Decreases the magnitude of the electric field outside the inter-electrode region	[3]
Mid-region width = gap between facing electrodes pair = Surrounding microchannel width	100	Higher signal strength and signal to noise ratio (SNR) with lowest noise. Elongation of the electrodes along the channel direction result in a higher current between the electrodes.	Required large amount of fluidic volume	Supplementary Figure S1, & Supplementary Table S3, [1, 2]
Sensing region cross sectional area	30×15	Overall sensitivity increases because smaller fluid volume occupied as compared to surrounding wider channel	Limited particle size detection compared to channel height	[1, 2]

Table S8. List of all meshing properties, number of degrees of freedom, solution time for four highly dense mesh settings.

Meshing Style	Mesh Elements	Number of degrees of freedom	Meshing time (s)	Average Element Quality	Max. Element size (µm)	Min. Element Size (µm)	Max. Element Growth rate
Normal	57137 domain<br>20069 boundary<br>2514 edge	93216	5.22	0.57	5.32	0.1	1.62
Fine	74393 domain<br>23426 boundary<br>2754 edge	118882	5.99	0.58	4.75	0.12	1.56
Finer	109863 domain<br>30369 boundary<br>3193 edge	171660	8.19	0.65	2.86	0.25	1.45
Extra Fine	230522 domain<br>51312 boundary<br>4176 edge	346354	10.22	0.66	1.95	0.09	1.36
Extremely Fine	7008784 domain<br>1048514 boundary<br>14093 edge	9908581	154	0.75	1	0.02	1.32

Table S9. List of all meshing properties, number of degrees of freedom, solution time for four highly dense mesh settings.

Meshing Style	Avg. Max. ΔE (kV/m)	σ_{noise} (kV/m)	SNR (dB)	Simulation Time (min)
Normal	235.9	13.6	24.77	11.5
Fine	219.6	12.5	24.83	10
Finer	577.7	14.2	32.18	16
Extra Fine	596.2	14.4	32.33	25
Extremely Fine	Simulation aborted due to extra long simulation time			more than 120

Table S10. Particle shape comparison with same volume

Particle (µm)	Sphere Dia=10	Ellipse-x 14×10×7.2	Ellipse-y 10×14×7.2
	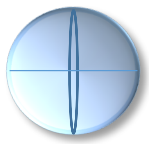	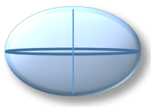	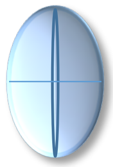
Length (µm)	10	14	10
S=width to length ratio	1	0.71	1.4
Volume (µm^3)	523.33	527.35	527.35
Amplitude (ΔE) (kV/m)	557	711	718
FWHM (µm)	26.9	27.4	26.2
R_A =width/height (mm/V)	0.0032	0.0025	0.0024
Amplitude ($\|E_2\|$) (kV/m)	38.2	23.8	34.2
FWHM (µm)	11.48	12.23	11.06
R_B =width/height (mm/V)	0.06	0.10	0.065

Table S11. Particle shape differentiation with different volume

Particle (µm)	Ellipse-x 10×1×10	Ellipse-x 10×5×10	Sphere Dia=10	Ellipse-y 10×15×10
	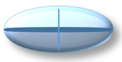	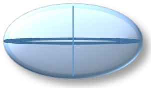	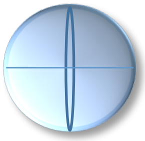	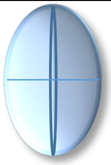
Length (µm)	10	10	10	10
S=width to length ratio	0.1	0.5	1	1.5
Volume (µm^3)	52.33	261.66	523.33	785
Amplitude (ΔE) (kV/m)	17.724	85.15	179.0	275.16
FWHM (µm)	23.73	23.11	23.24	22.79
R_A =width/height (mm/V)	0.09	0.018	0.0086	0.0055
Amplitude ($\|E_2\|$) (kV/m)	2.6	6.14	12.8	20.9
FWHM (µm)	12.40	12.81	10.49	10.19
R_B =width/height (mm/V)	0.95	0.41	0.16	0.097

Table S12. Particle shape differentiation in a mixture of particles containing 2 and 4 µm spherical particles and 3 µm (length or width) elliptical particle in IE-1 design

Particle (µm)	Sphere Dia=2	Sphere Dia=4	Ellipse-x 3×2×3	Ellipse-y 3×4×3
	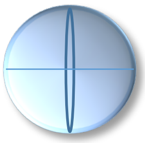	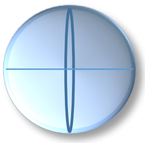	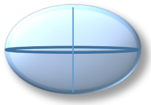	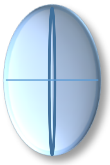
Length (µm)	2	4	3	3
S=width to length ratio	1	1	0.66	1.33
Volume (µm^3)	4.19	33.51	9.42	18.84
Amplitude (ΔE) (kV/m)	4.1	18.44	7.29	13.5
FWHM (µm)	39.59	25.88	23.14	22.65
R_A =width/height (mm/V)	0.64	0.09	0.21	0.11

Supplementary Figures

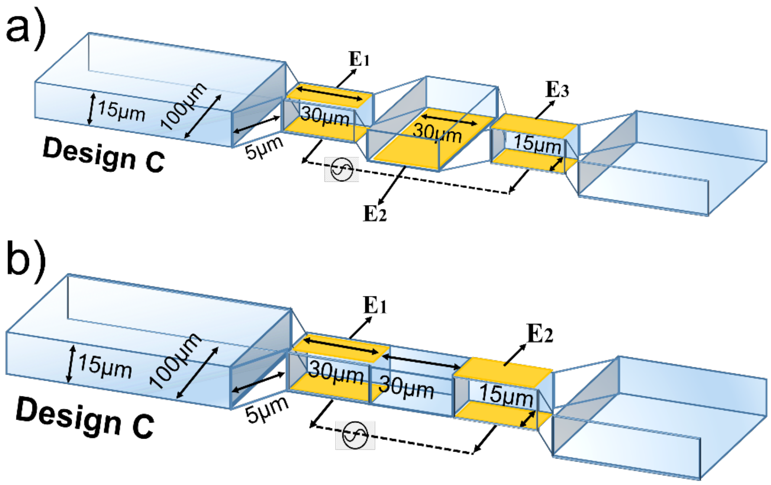

Figure S1. 3-D view of Top bottom microelectrode layout (Design C) with constricted microchannel geometry with a) broader central space b) and straight narrow central space. Figures are drawn in Microsoft PowerPoint 365.

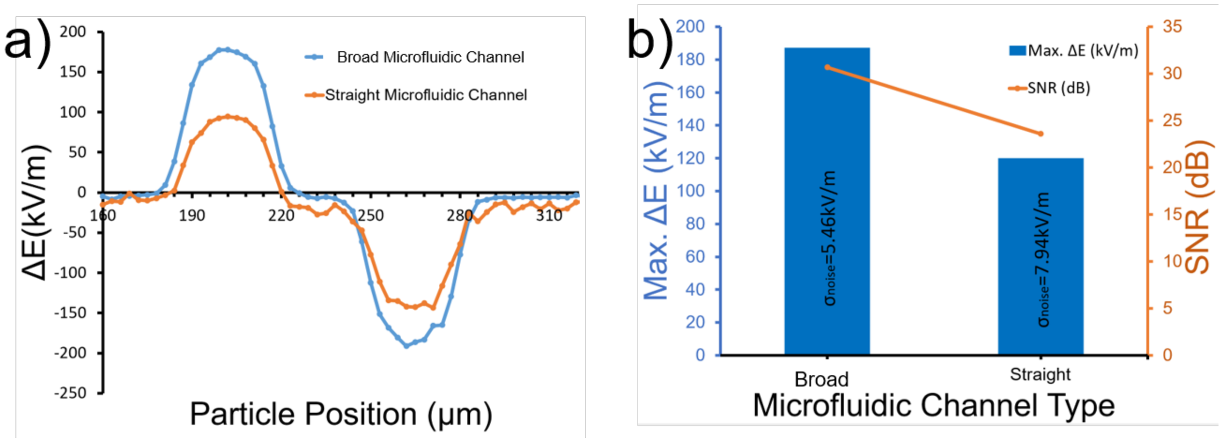

Figure S2. Electrical signal comparison in Design C electrode configuration with constricted broad central space produces higher signal strength and signal to noise ratio (SNR) with lowest noise as contrast to straight narrow central space.

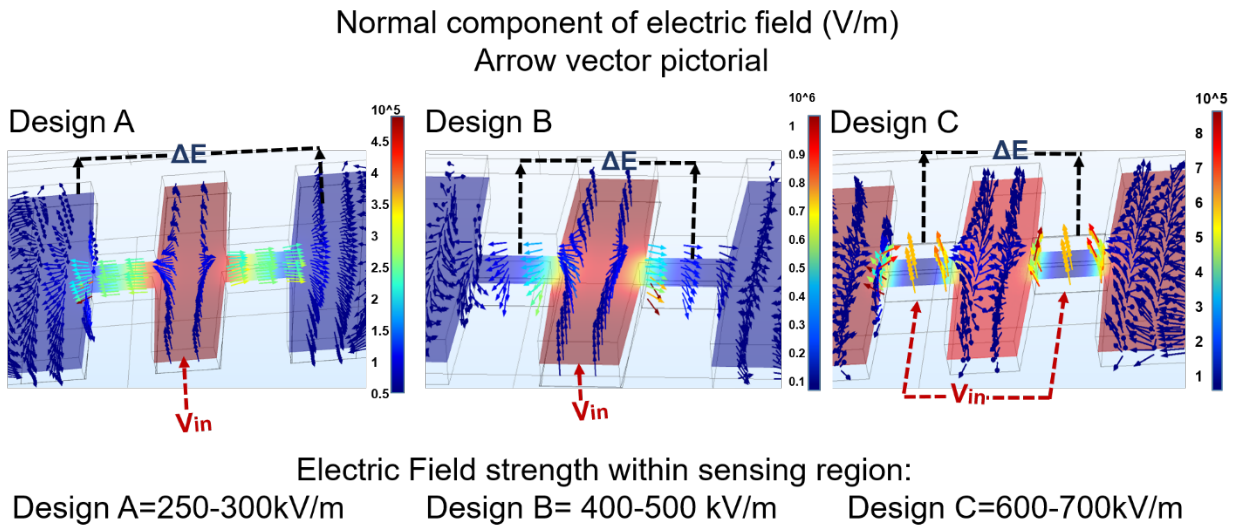

Figure S3: Normal component of Electric field arrow plots in the sensing region between electrodes for Design A, B & C. Figures are obtained using COMSOL Multiphysics version 5.3a (https://www.comsol.com/release/5.3a).

Design A is coplanar 3-electrode configuration in which voltage is applied to middle bottom electorde and signal is extracted from right and left hand side of sensing region bottom electrodes. The normal component of electric field arrow started from the middle bottom electrode towards both right and left positioned electrodes away from sensing region. Design B is modified layout in which signal is extracted from both bottom electrodes positioned under sensing region and middle ebottom electrode is electrically connected to external signal V_{in}. *The normal component of electric field arrow started from the middle bottom electrode towards both right and left positioned electrodes under the sensing region. Design C is conventional top-bottom electrode layout in while bottom both electrodes placed under sensing region are excited externally (*V_{in}*) and signal is obtained from both top electrodes positioned at top of sensing region. The normal component of electric field arrow directed from bottom towards top.*

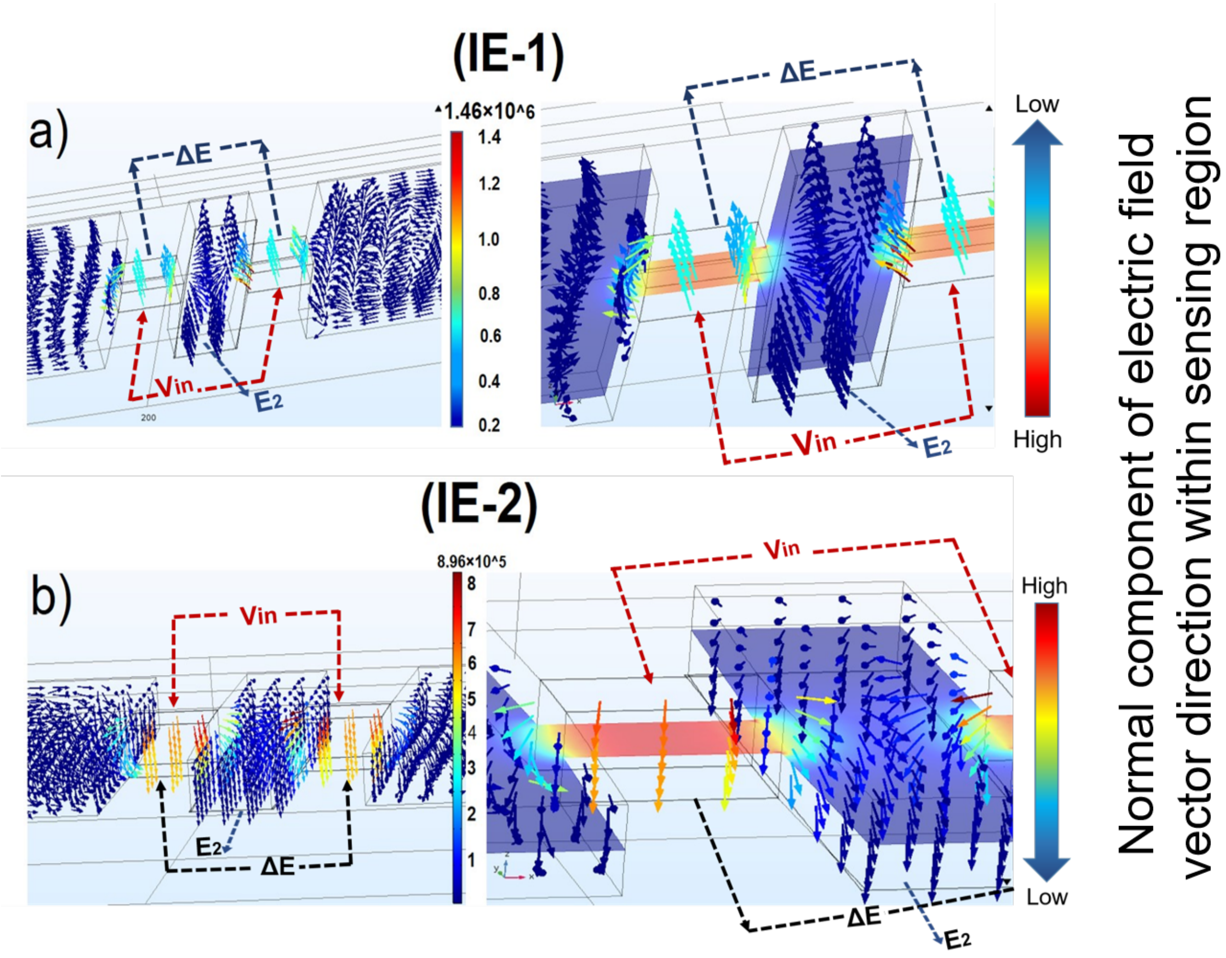

Figure S4: Normal component of Electric field distribution arrow plots in the sensing region between electrodes for both Integrated design IE-1 and IE-2 using COMSOL Multiphysics version 5.3a. a) In IE-1, both bottom electrodes in the aperature region are given an input signal of 10V, while output signal is collected from top electrodes and middle central electrode. b) In IE-2, input voltage is applied to top electrodes, and the output signal is collected from all three bottom electrodes. Figures are obtained using COMSOL Multiphysics version 5.3a (https://www.comsol.com/release/5.3a).

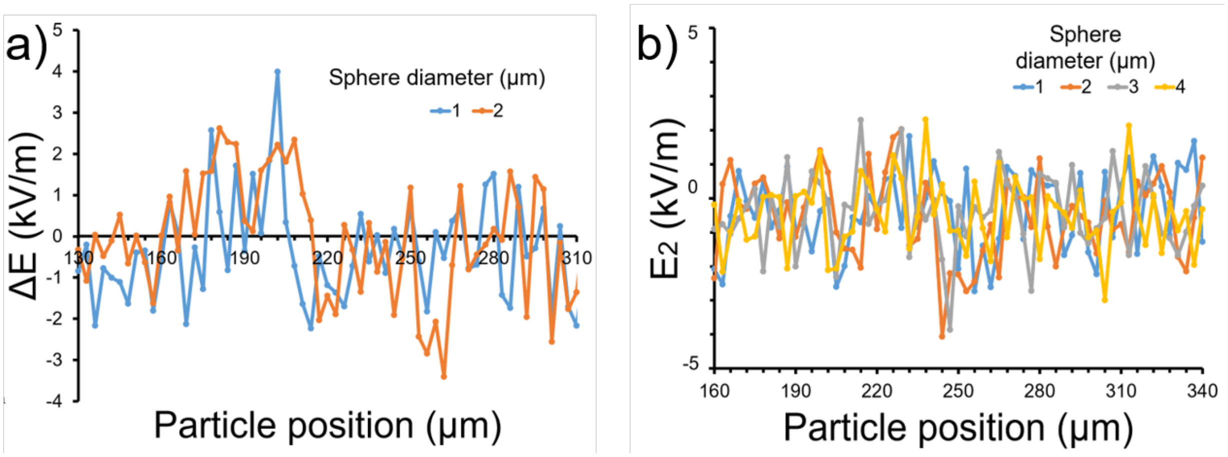

Figure S5. a & b) IE-1 design simulation results for ΔE and E_2 signals of spherical shape particles whose signal and noise is visually indistinguishable.

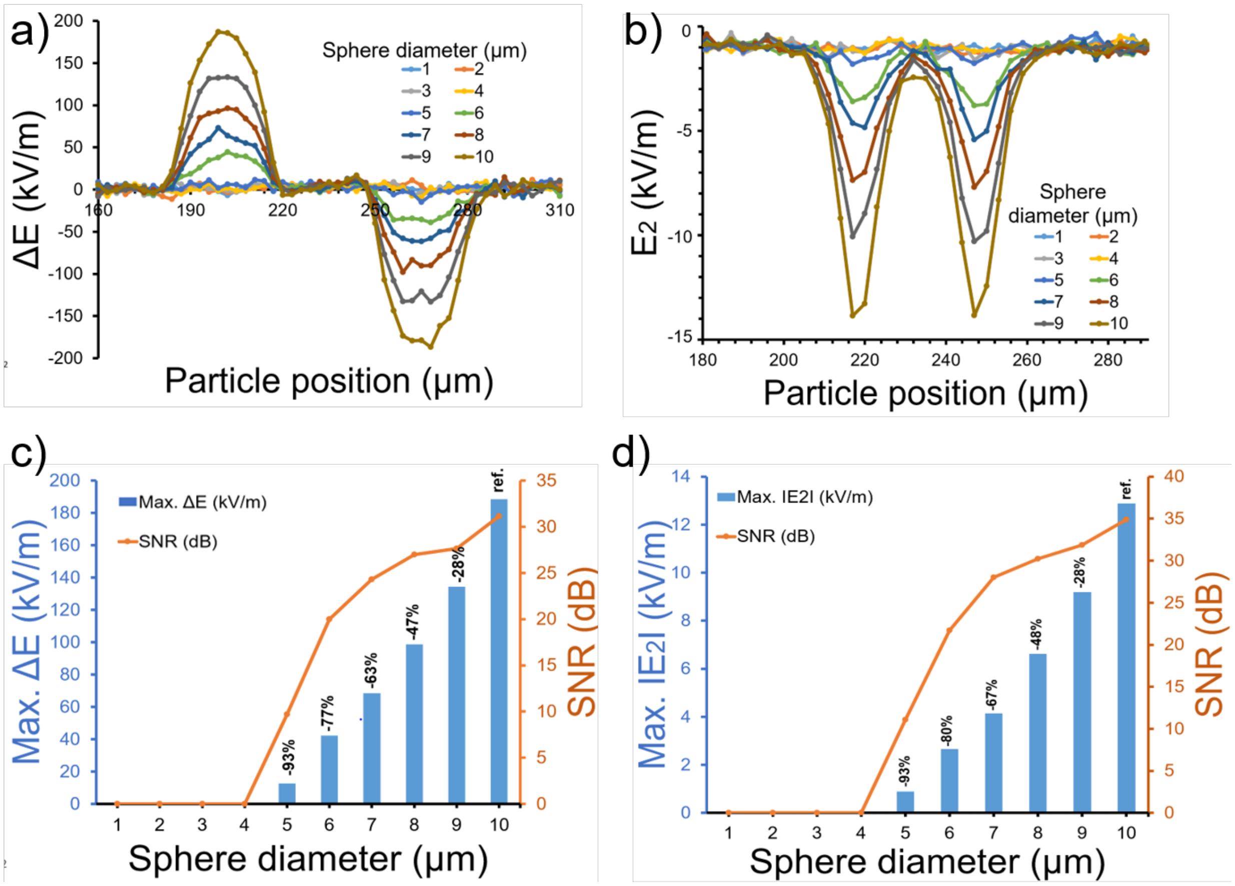

Figure S6. a & b) IE-2 design simulation results for ΔE and E_2 signals of a spherical shape particle versus diameter increment from 1 to 10 in μm, (c & d) Comparison of Maximum values of ΔE and $|E_2|$ and their resctive SNR in dB for spherical particle diameter ranges from 1 to 10 μm.

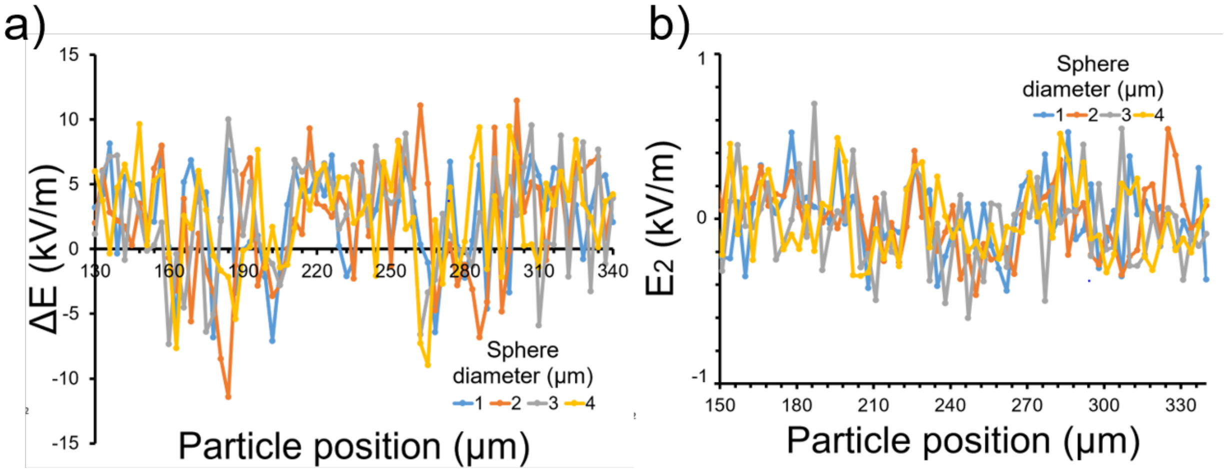

Figure S7. a & b) IE-2 design simulation results for ΔE and E_2 signals of spherical shape particles whose signal and noise is visually indistinguishable.

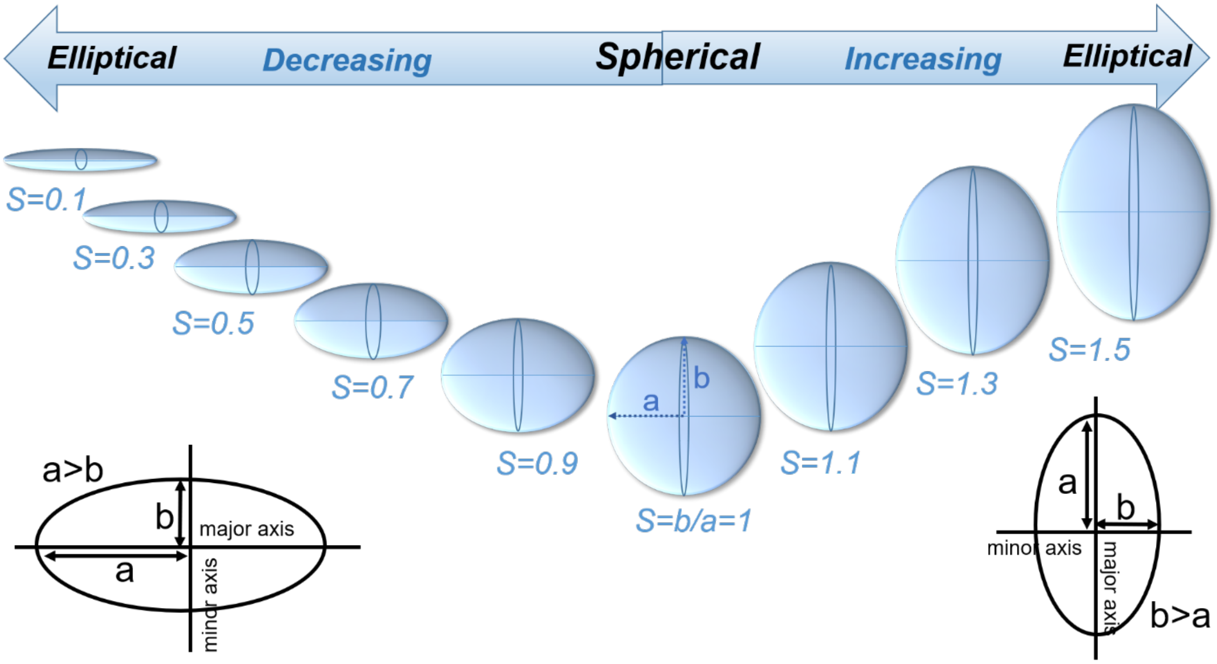

Figure S8. Gradual shape variation from spherical to ellipsoidal shape by wavering scaling factor based on the axis ratio with fixed minor axis, a = 5µm, changing scaling factor (S) from 0.1 to 1.5. On the right side, the minor axis b starts increasing as compared to a resulted into spherical to non-spherical and ended into ellipsoidal particle, while on the left side a minor axis is larger in size than b and partical deforms from spherical to non-spherical and culminates into ellipsoidal particle. Figures are drawn in Microsoft PowerPoint 365.

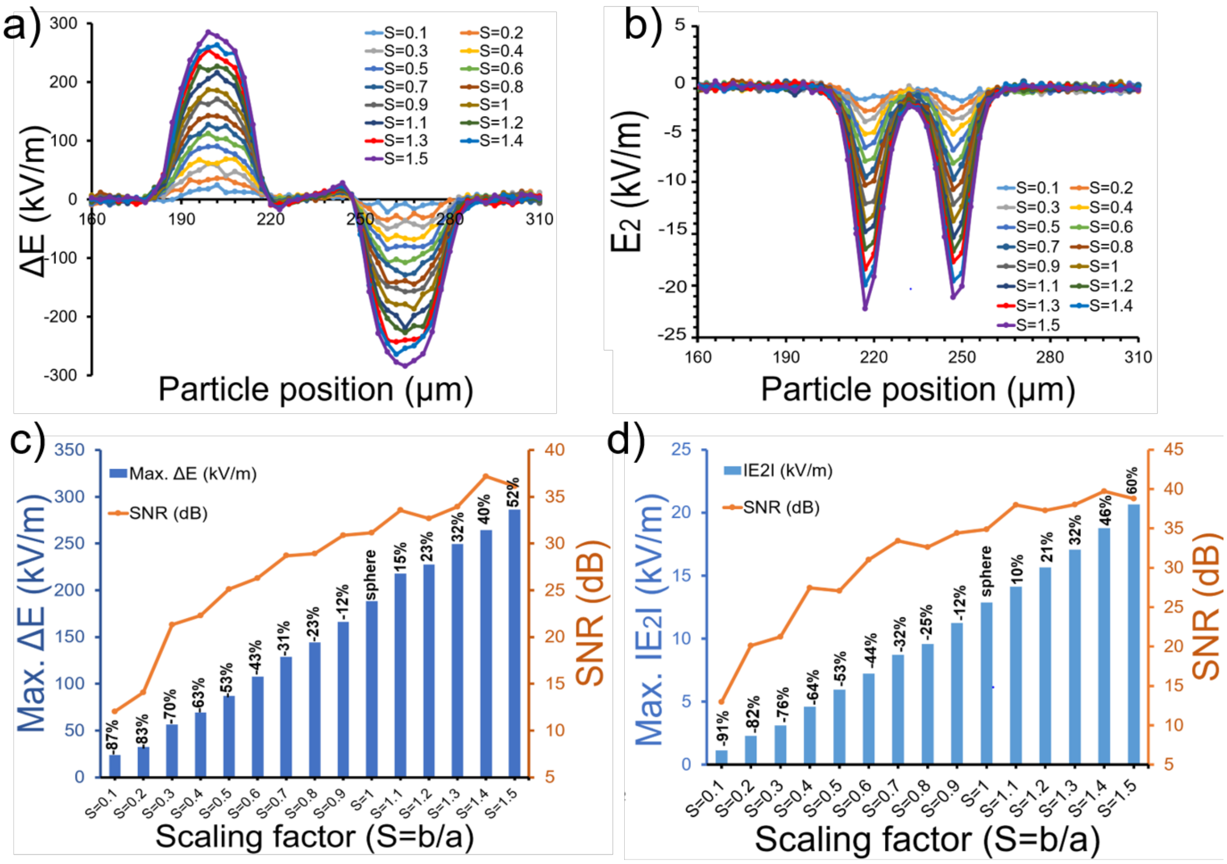

Figure S9. **IE-2**: Gradual change from spherical to non-spherical particle i.e. S = 0.1-1.5 (with a=5μm remains fixed) in integrated design IE-2. (a, b) Simulation of ΔE and $|E_2|$ signals for particle axis ratio, S=b/a from 0.1 to 1.5. (c,d) Comparison of maximum values of ΔE and $|E_2|$ and their resctive SNR in dB for particle shape ratio, S from 0.1 to 1.5.

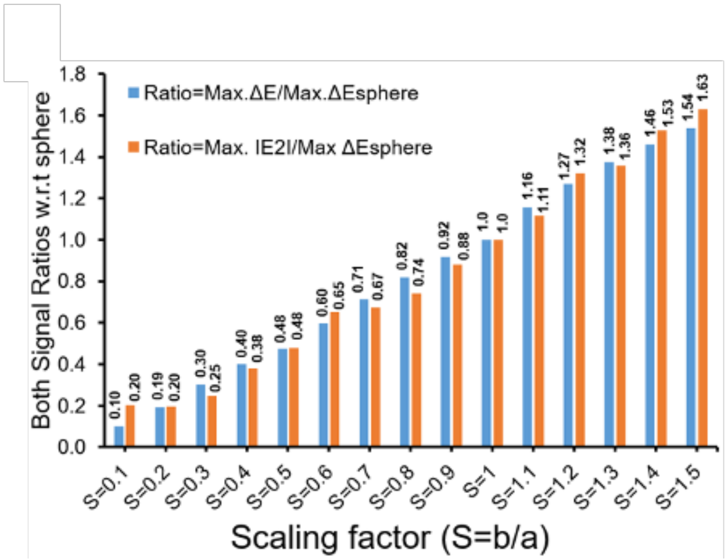

Figure S10. Maximum of both signal ratios (ΔE and │E₂│) with respect to sphere (S=1) in integrated design 2 (IE-2) illustrates a gradual change from spherical to non-spherical shape bioparticle. Scaling factor (S=b/a) increases from 0.1 to 1.5.

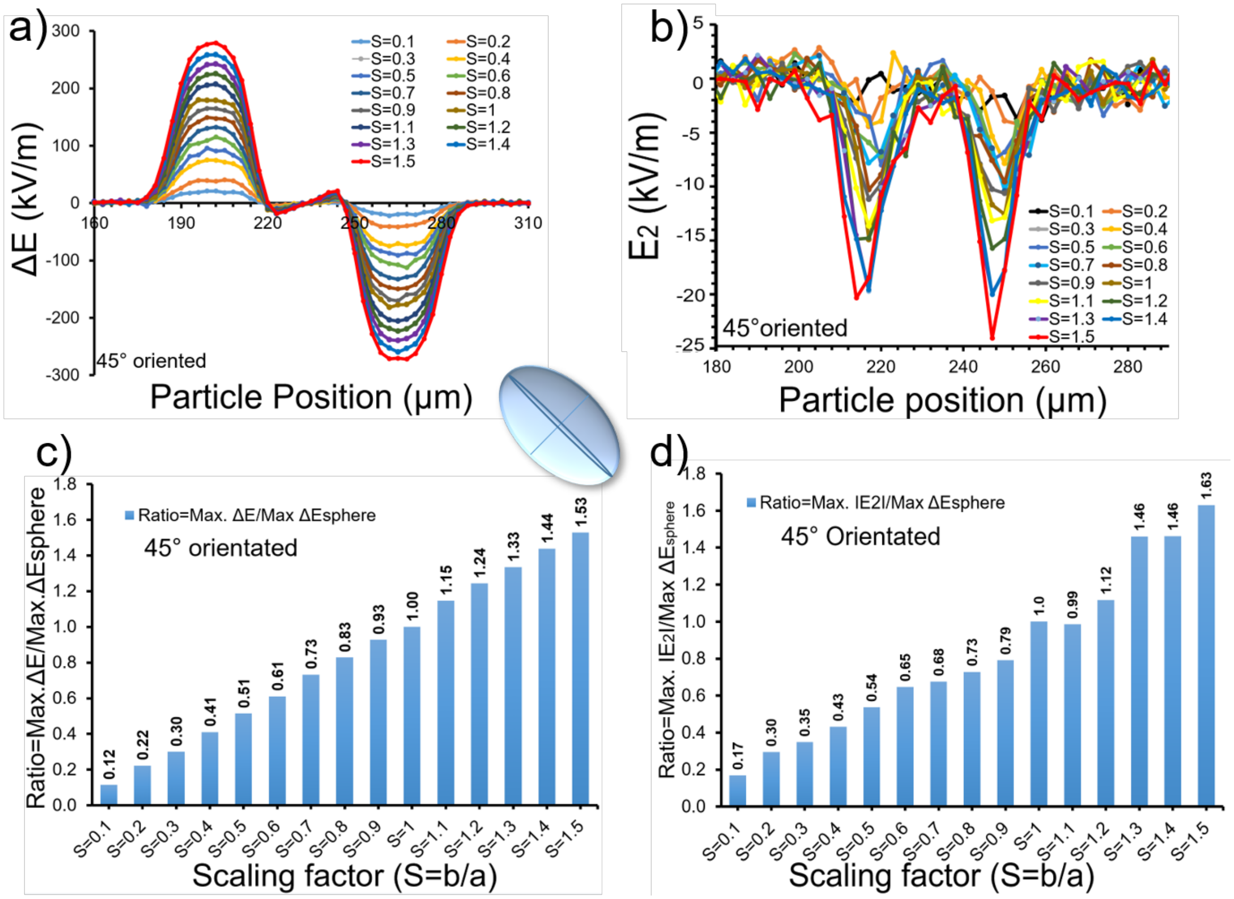

Figure S11. **IE-1 Design**: Gradual change from spherical to non-spherical bioparticle oriented at 45° with a=5µm remains fixed. a, b) Simulation of ΔE and $|E_2|$ signals for particle shape ratio, S=b/a from 0.1 to 1.5. c,d) Comparison of maximum values of ΔE and $|E_2|$ and their resctive SNR in dB for particle shape ratio, S from 0.1 to 1.5.

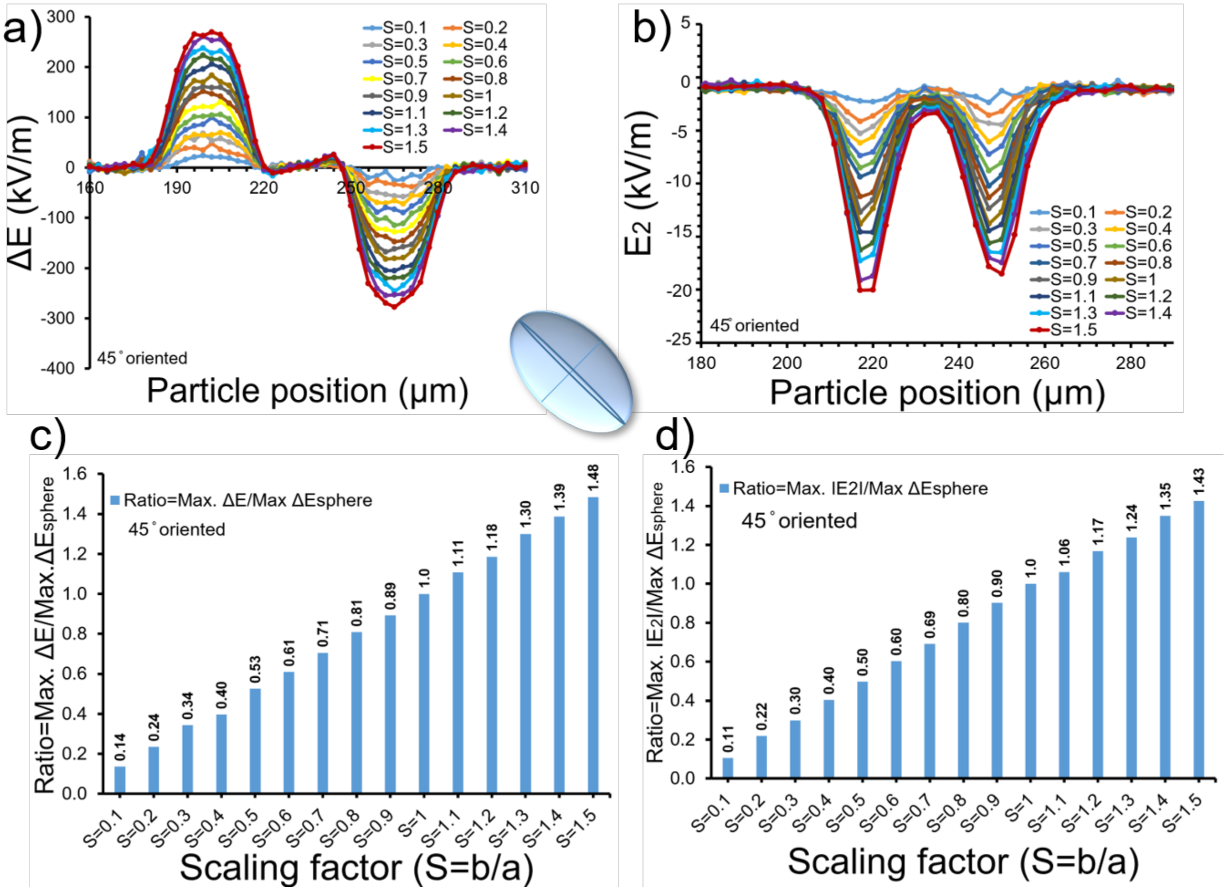

Figure S12. **IE-2 Design**: Gradual change from spherical to non-spherical shape bioparticle oriented at 45° with a=5μm remains fixed. a, b) Simulation of ΔE and E_2 signals for particle shape ratio, S=b/a from 0.1 to 1.5. c,d) Comparison of Maximum values of ΔE and $|E_2|$ and their resctive SNR in dB for particle shape ratio, S from 0.1 to 1.5.

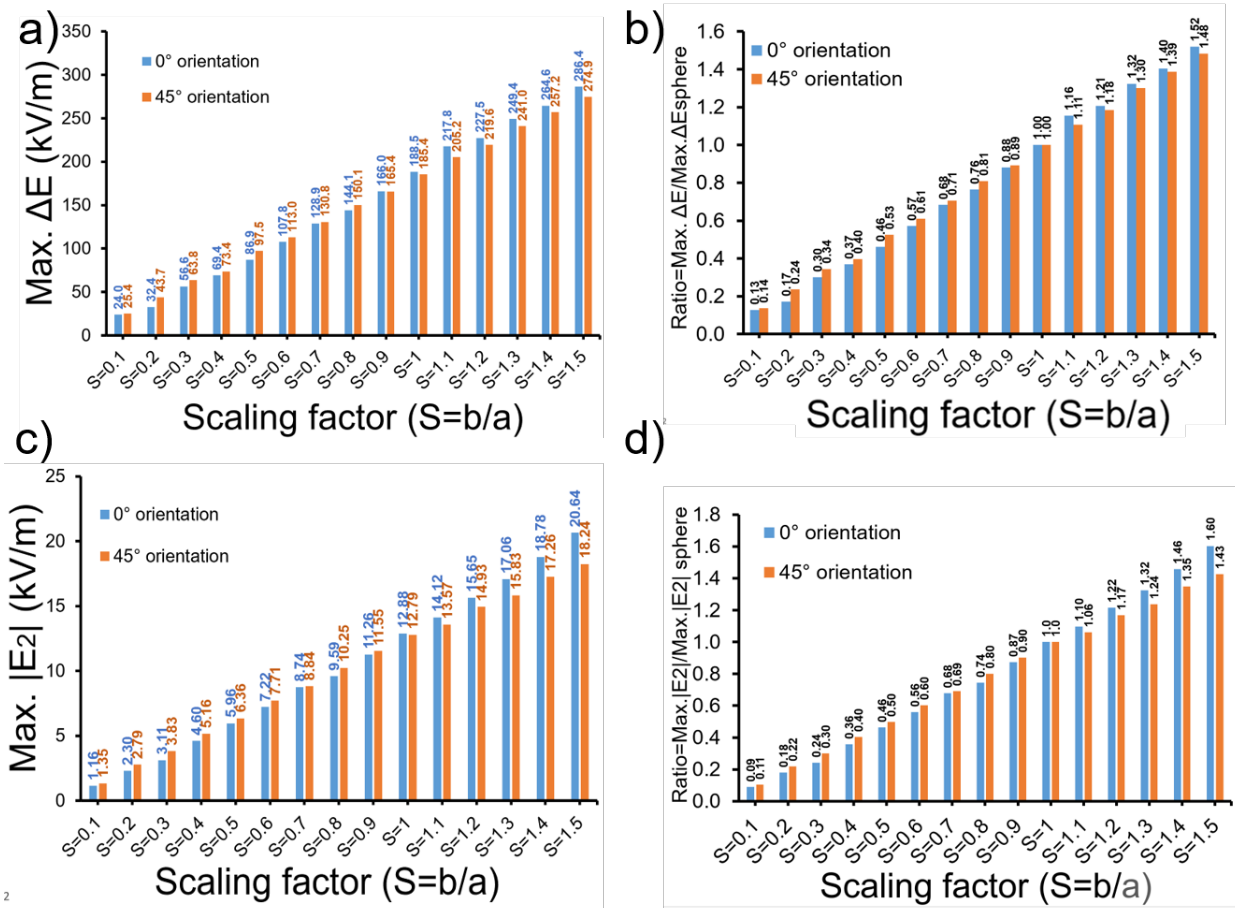

Figure S13. Comparison of spherical and non-spherical particle signal variation with respect to orientation (0-degree and 45-degree) within integrated design **IE-2**. a & c) Maximum of ΔE and $|E_2|$ signal with respect to scaling factor (S=1) increases from 0.1 to 1.5 illustrates a gradual change from spherical to non-spherical shape bioparticle, b & d) Maximum of ΔE & $|E_2|$ signal ratio in regard to sphere peak value at zero and 45-degree oriented particle.

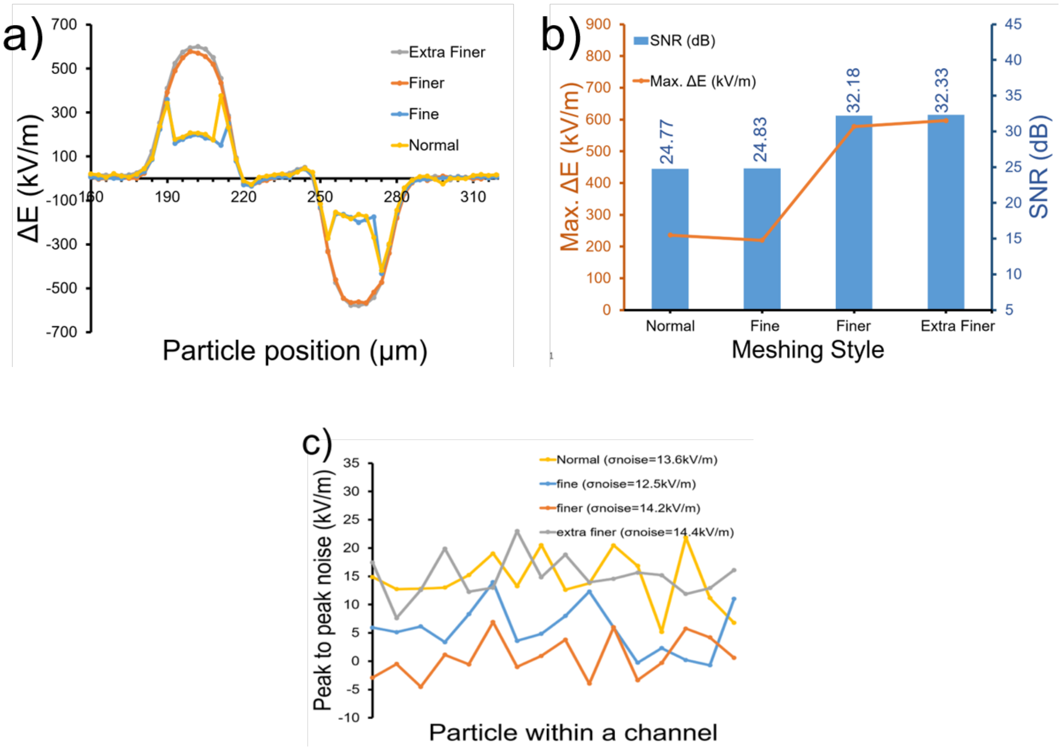

Figure S14. a) Output signal ΔE from IE-1 relative to particle position in the channel at three mesh settings fine, finer, and extra finer, b) Electrical signal comparison in three mesh settings. Finer and extra finer mesh settings show promising electric signal strength and signal to noise ratio as compared to fine mesh settings.

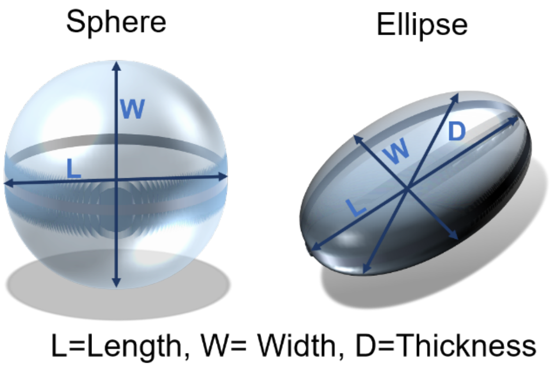

Figure S15. Spherical and Elliptical Particle Geometry

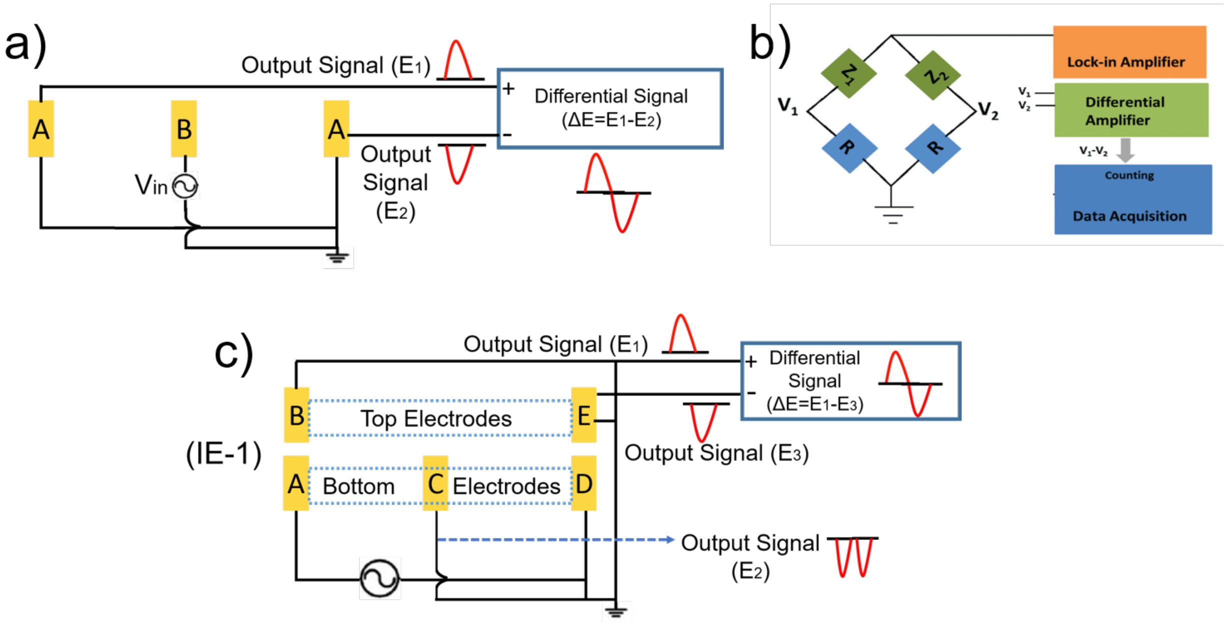

Figure S16: Electrical setup for microelectrodes a) The electrical measurement setup for data acquisition in standard coplanar electrode configuration. The input signal (V_{in}) is applied to the middle electrode (B) surface and ground the other two electrodes A & B. Differential signal ΔE is obtained by taking the difference of two signals E_1 & E_2 from the surface of A & B electrodes. b) Experimental setup for data acquisition from coplanar microelectrodes in practical word experimentation [Lab Chip, 2014, 14, 1469-1476]. The lock-in-amplifier (LIA) is used to inject the input signal to the electrodes and the resulting signal is acquired using a Wheatstone bridge. Metering is done by measuring the potential drop across R. However, for particle counting the differential signal is used. c) The electrical measurement setup for data acquisition in our integrated multiplanar electrode configuration, IE-1. The input signal (V_{in}) is applied to the electrode A & D surface and ground the other all electrodes B, C & E. Differential signal ΔE is obtained by taking the difference of two signals E_1 & E_3 obtained from the surface of B & E electrodes, and E_2 is signal is directly acquired from the middle electrode (C) surface.

Table S13. The comparative study of current and previous studies. The size, shape and cell classification in single cell cytometry has been tabulated in detail. Present study offers higher signal strength & SNR for a biological cell inclusive of particle type and shape differentiation.

**Articles**	Our Work	[6][1]	[7][2]	[8][3]	[9][4]	[10][5]
**Study Approach**	Computational	Computational	Computational+ Experimental	Experimental	Computational + Experimental	Experimental
**Electrode geometry**	Coplanar + Planar 5-Electrode Setup	3-D Electrodes, Micro sieve fabrication	Coplanar 3-Electrode Setup	Liquid Electrodes+ 8-Electrode Setup	TOP-Bottom Electrode setup (5-pairs),	Coplanar 3-Electrode (Open & closed setup)
**Particle Type & size**	1-10µmBead+ Biological Cell	Air filled cell of 5µm cell	RBC & Leukemia cells Beads (3 &6µm)	RBC, Yeast cells, Beads (5,6,7µm)	RBCs, Beads (5.49, 7.18µm)	Bead (9µm & 2.8µm)
**Particle Shape**	Spherical+ Elliptical	spherical	Spherical	Spherical	Spherical	Spherical
**Electrode Dimension**	30µm×15/5µm width × Gap	20µm ×20µm width × Gap	10µm ×10µm width × Gap	20µm ×20µm width × Gap	30µm ×10µm width × Gap	40/20µm ×50µm width × Gap
**Particle Size discrimination**	Max. SNR~40.2 dB for Biological Cell	~ 5µm air-filled cell	Size based Sorting~3µm.	~ 5µm & Max. SNR~30dB	Max. SNR~23.4dB for RBC, Max. SNR~23.2dB for 6µm Bead.	Max. SNR~18dB for 2.8µm Bead
**Particle Type Classification**	Yes, Provides clear differentiation of polystyrene bead from biological cells and among different conductivity cells.	No	Yes	Yes	Yes	No
**Shape Differentiation**	Yes, provides discriminative values for Spherical & Elliptical shape particle	No	No	No, Need improvement to discriminate cell shape	No	No
**Channel Height**	15 µm	100µm	10 µm	30 µm	40 µm	30 µm
**Limitation**	Experimental data will be provided in near future.	Micro sieve fabrication, & complex alignment. Experimental proof is required	Differentiation based on volumetric difference	Machine Learning Approach is required	Event detection Algorithm is required for enhanced sensitivity	Lack characterizatio n of cellular properties particles

Articles	[11][6]	[12][7]	[13][8]	[14][9]	[15][10]	[16][11]
**Study Approach**	Computational+ Experimental	Computational	Experimental	Computational+ Experimental	Computational+ Experimental	Experimental
**Electrode geometry**	Complex Coplanar electrode setup	Tetrapolar coplanar & parallel Electrode setup	Pair of Coplanar electrode	Pair of Top-Bottom Electrode Used Insulating Fluid of Oil to focus particles.	2-Coplanar Electrode setup. Used Highly conductive solution	Floating coplanar Electrodes+ pair of parallel electrodes
**Particle Type & size**	Beads (5,8μm) RBCs	PMMA (ε=2.6) Length=70μm	Beads & Yeast 3μm, *S. aureus/ S. albicans* (0.8μm)	Bead (1 & 2μm) *E. Coli*	Bead (1 & 2μm) *E. Coli*	Bead (3 & 6μm)
**Particle Shape**	Spherical	Cube	Spherical	Spherical & Rod	Spherical & Rod	Spherical
**Electrode Dimension**	20μm ×20μm width × Gap	60μm ×60μm width × Gap	10μm ×5μm width × Gap	20μm ×40μm width × Gap	5μm ×5μm 10μm×10μm width × Gap	15μm ×18μm width × Gap
**Particle Size discrimination**	~5μm	~ 70μm	~ 3μm,	~ 1 & 2μm	Max. SNR~20dB for 2μm Bead.	~ 3μm
**Particle Type Classification**	Yes	No	Yes	Couldn't well distinguish between *E. Coli* and 2μm bead	Yes	No
**Shape Differentiation**	No	No	No	No	No	No
**Channel Height**	20 μm	50 μm	7 μm	30 μm	10 μm	-------
**Limitation**	Lower sensitivity	Lack characterization of cellular properties particles	Signal amplitude overlaps for two types of bacteria even at high conductive buffer solution	Use oil for restriction of electric field lines to enhance sensitivity	Cumbersome device fabrication due to smallest electrode and channel geometry	Too many extra fabrication steps required

**Articles**	[17][12]	[1][13]	[18][14]	[2][15]	[19][16]
**Study Approach**	Experimental	Computational+ Experimental	Experimental	Computational+ Experimental	Experimental
**Electrode geometry**	Optical focusing with Top-Bottom Electrode	Coplanar Electrode layout with constricted channel	Separately compare Coplanar and parallel electrode configuration	2-electrode Coplanar layout	2-electrode Coplanar layout
**Particle Type & size**	Bead of 2 & 3μm, *E. Coli* & *B. subtilis*	Bead 0.5, 1 & 2μm	Bead of 1 & 2μm, *E. Coli* & *S. aureus*	8μm bead	5 & 8μm bead SU-8 Rods (4×4×8μm)
**Particle Shape**	Spherical & Rod	Spherical	Spherical & Rod	Spherical	Spherical+ Rod
**Electrode Dimension**	18μm×18μm width × Gap	10μm ×5μm width × Gap	10μm ×16μm width × Gap	50μm ×50/150μm width × Gap	10μm ×10μm width × Gap
**Particle Size discrimination**	~ 2μm	~ 0.5, 1 & 2μm	Slight Phase signal differentiation for 1 & 2μm bead	≤8μm	~ 5μm
**Particle Type Classification**	Unable to differentiate between bead and bacteria	No	Yes	No	No
**Shape Differentiation**	No	No	Overlap Phase Signal	No	Budding Yeast shape monitoring
**Channel Height**	10 μm	5 μm	10 μm	50 μm	12 μm
**Limitation**	Additional DEP electrodes for particle focusing & circuitry to improve sensitivity	Sensor sensitivity decreases in the presence of a biological cell which is not considered here.	System lack sensitivity due to Inaccurate Bacterial Count. Phase signal also changes in PBS/conductive solution	No data for small particles, and biological cells	Separate 3-pair of electrodes for focusing is required

References

1. C. H. Clausen, G. E. Skands, C. V. Bertelsen and W. E. Svendsen, *Micromachines*, 2015, **6**, 110-120.
2. J. Cottet, A. Kehren, H. van Lintel, F. Buret, M. Frénéa-Robin and P. Renaud, *Microfluidics and nanofluidics*, 2019, **23**, 11.
3. T. Sun, N. G. Green, S. Gawad and H. Morgan, *IET nanobiotechnology*, 2007, **1**, 69-79.
4. D. Spencer and H. Morgan, *Lab on a Chip*, 2011, **11**, 1234-1239.
5. N. N. Watkins, S. Sridhar, X. Cheng, G. D. Chen, M. Toner, W. Rodriguez and R. Bashir, *Lab on a Chip*, 2011, **11**, 1437-1447.
6. Y. Demircan Yalcin and R. Luttge, *Journal of Vacuum Science & Technology B, Nanotechnology and Microelectronics: Materials, Processing, Measurement, and Phenomena*, 2020, **38**, 063202.
7. I. Bilican, M. T. Guler, M. Serhatlioglu, T. Kirindi and C. Elbuken, *Sensors and Actuators B: Chemical*, 2020, **307**, 127531.
8. C. Honrado, J. S. McGrath, R. Reale, P. Bisegna, N. S. Swami and F. Caselli, *Analytical and bioanalytical chemistry*, 2020, 1-11.
9. F. Caselli and P. Bisegna, *IEEE Transactions on Biomedical Engineering*, 2015, **63**, 415-422.
10. S. Emaminejad, K.-H. Paik, V. Tabard-Cossa and M. Javanmard, *Sensors and Actuators B: Chemical*, 2016, **224**, 275-281.
11. S. Gawad, L. Schild and P. Renaud, *Lab on a Chip*, 2001, **1**, 76-82.
12. M. Hantschke and I. F. Triantis, *IEEE Sensors Journal*, 2020.
13. Z. Zhang, M. Gong, X. Xie, Y. Du, Z. Cheng and W. Zhou, 2020.
14. C. Bernabini, D. Holmes and H. Morgan, *Lab on a Chip*, 2011, **11**, 407-412.
15. M. T. Guler and I. Bilican, *Sensors and Actuators A: Physical*, 2018, **269**, 454-463.
16. L. I. Segerink, A. J. Sprenkels, J. G. Bomer, I. Vermes and A. van den Berg, *Lab on a Chip*, 2011, **11**, 1995-2001.
17. N. Haandbæk, S. C. Bürgel, F. Heer and A. Hierlemann, *Lab on a Chip*, 2014, **14**, 3313-3324.
18. C. H. Clausen, M. Dimaki, C. V. Bertelsen, G. E. Skands, R. Rodriguez-Trujillo, J. D. Thomsen and W. E. Svendsen, *Sensors*, 2018, **18**, 3496.
19. X. Xie, Z. Zhang, X. Ge, X. Zhao, L. Hao, Z. Cheng, W. Zhou, Y. Du, L. Wang and F. Tian, *Analytical chemistry*, 2019, **91**, 13398-13406.
